# A novel mechanism for macrophage pyroptosis in rheumatoid arthritis induced by Pol β deficiency

**DOI:** 10.1038/s41419-022-05047-6

**Published:** 2022-07-06

**Authors:** Lili Gu, Yuling Sun, Ting Wu, Ge Chen, Xiaojun Tang, Lianfeng Zhao, Lingfeng He, Zhigang Hu, Lingyun Sun, Feiyan Pan, Zhimin Yin, Zhigang Guo

**Affiliations:** 1 Jiangsu Key Laboratory for Molecular and Medical Biotechnology, College of Life Sciences, Nanjing Normal University, 1 Wen Yuan Road, Nanjing, 210023 China; 2 Department of Rheumatology and Immunology, the Affiliated Drum Tower Hospital of Nanjing University Medical School, Nanjing, 210008 China

**Keywords:** Immune cell death, Base excision repair

## Abstract

Rheumatoid arthritis (RA) is a chronic and inflammatory autoimmune disease. Macrophage pyroptosis, a proinflammatory form of cell death, is critically important in RA; however, the detailed mechanism underlying pyroptosis induction is not yet well understood. Here, we report that DNA polymerase β (Pol β), a key enzyme in base excision repair, plays a pivotal role in RA pathogenesis. Our data shows that Pol β expression is significantly decreased in peripheral blood mononuclear cells (PBMCs) from active RA patients and collagen-induced arthritis (CIA) mice, and Pol β deficiency increases the incidence of RA, macrophage infiltration, and bone destruction in CIA mouse models. In vitro, experiments showed that Pol β deficiency exacerbated macrophage pyroptosis induced by LPS plus ATP, while overexpression of Pol β inhibited macrophage pyroptosis. Further characterization revealed that Pol β knockout resulted in DNA damage accumulation and cytosolic dsDNA leakage, which activated the cGAS-STING-NF-κB signaling pathway and upregulated the expression of NLRP3, IL-1 β, and IL-18. In conclusion, our findings clarify the influence of Pol β on the development of RA and provide a detailed explanation for the STING-NF-κB pathway to induce macrophage pyroptosis.

## Introduction

As a chronic inflammatory joint disease, rheumatoid arthritis (RA) is characterized by synovial inflammation, hyperplasia, and autoantibody production and can cause cartilage and bone damage, as well as disability [1, 2]. Rheumatoid arthritis may involve a complex interplay of genotype, environmental triggers, and chance. A variety of innate effector cells, including macrophages, mast cells, natural killer cells, and neutrophils, are involved in RA pathogenesis [1]. Although the etiology of RA remains elusive, it is believed that macrophages play important roles in RA pathogenesis. There are a large number of macrophages in the cartilage tissue and synovial tissue of RA patients, and the number of macrophages has a certain correlation with the degree of joint injury and clinical symptoms [3]. Since macrophages generate cytokines that enhance inflammation and contribute to the destruction of cartilage and bone, these cells are of critical importance in RA.

Pyroptosis, one form of programmed cell necrosis, is emerging as a general innate immune mechanism and is characterized by rapid cytomembrane rupture and the release of proinflammatory cytokines, particularly IL-1β and IL-18 [4, 5]. Previous studies have shown that RA monocytes are prone to caspase-1-dependent pyroptosis, including promoting NLRP3 inflammasome overactivation, GSDMD cleavage, and caspase-1-dependent secretion of IL-1β and IL-18 [6]. Additionally, emerging evidence has suggested that NLRP3 expression and NLRP3-mediated IL-1β secretion are increased in whole blood cells from active RA patients upon stimulation via TLR3 and TLR4 [7]. The finding also indicates that A20 deficiency in macrophages significantly enhances NLRP3 inflammasome-mediated caspase-1 activation and IL-1 β secretion, which contributes to the pathology of rheumatoid arthritis in vivo [8]. Hence, these studies showed that monocyte pyroptosis contributes to the pathogenesis of RA. However, the intrinsic mechanism of pyroptosis induction in RA is still unclear.

DNA polymerase beta (Pol β) is a key enzyme in base excision repair (BER) [9]. In addition to functioning in DNA repair, Pol β was also reported to participate in other physiological activities, such as tumor growth, cell transformation, and tumorigenesis [10, 11]. Our studies have reported that Pol β modulates cancer progression by enhancing CDH13 expression by promoting DNA demethylation [12]. Moreover, Pol β deficiency leads to neurodegeneration and exacerbates Alzheimer’s disease phenotypes [13]. Double inactivation of Pol β and ataxia telangiectasia mutated (ATM) results in cerebellar ataxia [14]. Recently, a Pol β mutation (Y265C) has been reported to be an underlying cause of systemic lupus erythematosus (SLE) [15], which indicates that Pol β may be associated with the inflammatory response. However, whether Pol β is involved in other immune diseases and the underlying mechanism is still unclear.

Accordingly, the present study was designed to investigate the effects of Pol β on RA. We demonstrated that Pol β deficiency significantly exacerbated disease severity. Moreover, the functions of Pol β in regulating RA are achieved by inhibiting macrophage pyroptosis. Further studies indicated that the regulation of macrophage pyroptosis by Pol β was dependent on the cGAS-STING-NF-κB pathway. Overall, this study demonstrates the important function of Pol β in RA pathogenesis and provides molecular insight into the cause of DNA damage-mediated innate immune disease.

## Results

### Pol β levels are decreased in active RA patients and collagen-induced arthritis (CIA) mice

**Fig. 1:**
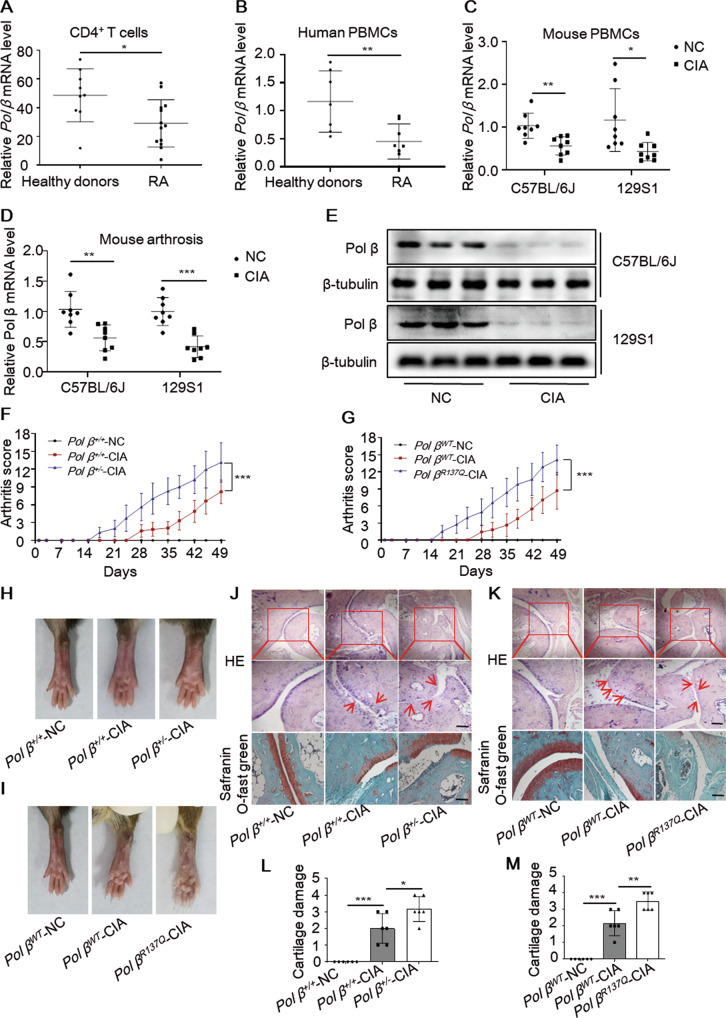
Pol β deficiency exacerbates disease severity in CIA mice. **A**, **B** Pol β expression in CD4^+^ T cells (GEO database: GSE56649) and PBMCs from active RA patients and healthy donors. All data are means ± SD from at least six independent samples. *P < 0.05, **P < 0.01. Student’s *t*-test (**A**, **B**). **C** qRT-PCR analysis of Pol β mRNA expression in the PBMCs of CIA mice. **D** The levels of Pol β mRNA in CIA mice’s ankles were measured by qRT-PCR. Data present mean ± SD from at least 6 mice, *P < 0.05, **P < 0.01, ***P < 0.001, two-way ANOVA. **E** The levels of Pol β protein in CIA mice’s ankles were measured by Western blotting. Data are representative blot of three independent experiments. Pol β deficiency exacerbates disease severity in CIA mice. **F**, **G** The clinical arthritic scores of CIA mice. Data present mean ± SD from at least 6 mice, ***P < 0.001, two-way ANOVA. **H**, **I** Macroscopic images of CIA mice, images were taken on Day 49 before the mice were sacrificed. **J**, **K** Histopathological analysis of CIA mice. Ankle joints were stained with H&E. Arrow indicates inflammatory cell infiltration. Scale bar, 500 μm. **L**, **M** The cartilage damage of CIA mice. Data present mean ± SD from at least 6 mice, *P < 0.05, **P < 0.01, ***P < 0.001, one-way ANOVA.

As a key enzyme in the BER pathway, Pol β mutations lead to reduced BER efficiency and genomic instability, resulting in the occurrence of autoimmune diseases and chronic inflammation [15, 16]. To assess whether Pol β is involved in the occurrence of RA, we first took the advantage of the availability of the public RNA-seq data from the Gene Expression Omnibus (GEO) and compared the expression of Pol β in T cells between active RA patients and healthy donors (GEO: GSE56649) [17]. The data showed that Pol β mRNA level was lower in active RA patients than in healthy donors (Fig. 1A). We further isolated PBMCs from active RA patients and healthy donors and measured Pol β mRNA level (Table S2). It was shown that Pol β was significantly down-regulated in PBMCs from RA patients (Fig. 1B). These results indicate that Pol β may be associated with RA occurrence. To further clarify the relationship between Pol β and RA development, we established CIA mouse models by injecting chicken type II collagen (Fig. S1) and examined the mRNA and protein levels of Pol β in the ankles of mice by qRT-PCR and Western blotting, respectively. Our data showed that Pol β mRNA and protein levels were dramatically decreased in CIA mice compared with mice in the control group (Fig. 1C–E). These results suggest that Pol β expression correlates with rheumatoid arthritis.

### Pol β deficiency exacerbates disease severity in CIA mice

To investigate whether Pol β is involved in the occurrence of RA, we created *Pol β*^*+/−*^ mice by CRISPR/Cas9-mediated genome engineering [18] (Fig. S2) and constructed a *Pol β*^*R137Q*^ mouse model using targeted gene disruption [19, 20]. Actually, no obviously visible phenotype was observed in both kinds of transgenic mice, however, the inflammatory factors (IL-1β, IL-18, IFNα, IFNβ) in different tissues were detected upregulated with Pol β deficiency (Fig. S3). Considering the classical function of Pol β in DNA repair, we then wondered whether the role of Pol β was specific for stress conditions. To better clarify the important role of Pol β in RA, we established CIA models on the basis of transgenic mice. Body weight and arthritis scores were measured twice per week during the study. We found that body weight was significantly reduced in *Pol β*^*+/−*^ mice and *Pol β*^*R137Q*^ mice (Fig. S4), while arthritis scores (Fig. 1F, G) and paw swelling were significantly increased (Fig. 1 H, I). Moreover, paw swelling occurred earlier in *Pol β*^*+/−*^ mice and *Pol* *β*^*R137Q*^ mice than in *WT* mice. Histopathological analysis and safranin O/ fast green staining also showed that inflammatory cell infiltration, bone destruction, and cartilage injury were more serious in *Pol β*^*+/−*^-CIA mice and *Pol* *β*^*R137Q*^-CIA mice than in *WT* mice (Fig. 1J–M). Therefore, our data showed that Pol β deficiency exacerbated disease severity in CIA mice.

### Pol β deficiency exacerbates the inflammatory response and macrophage pyroptosis

**Fig. 2:**
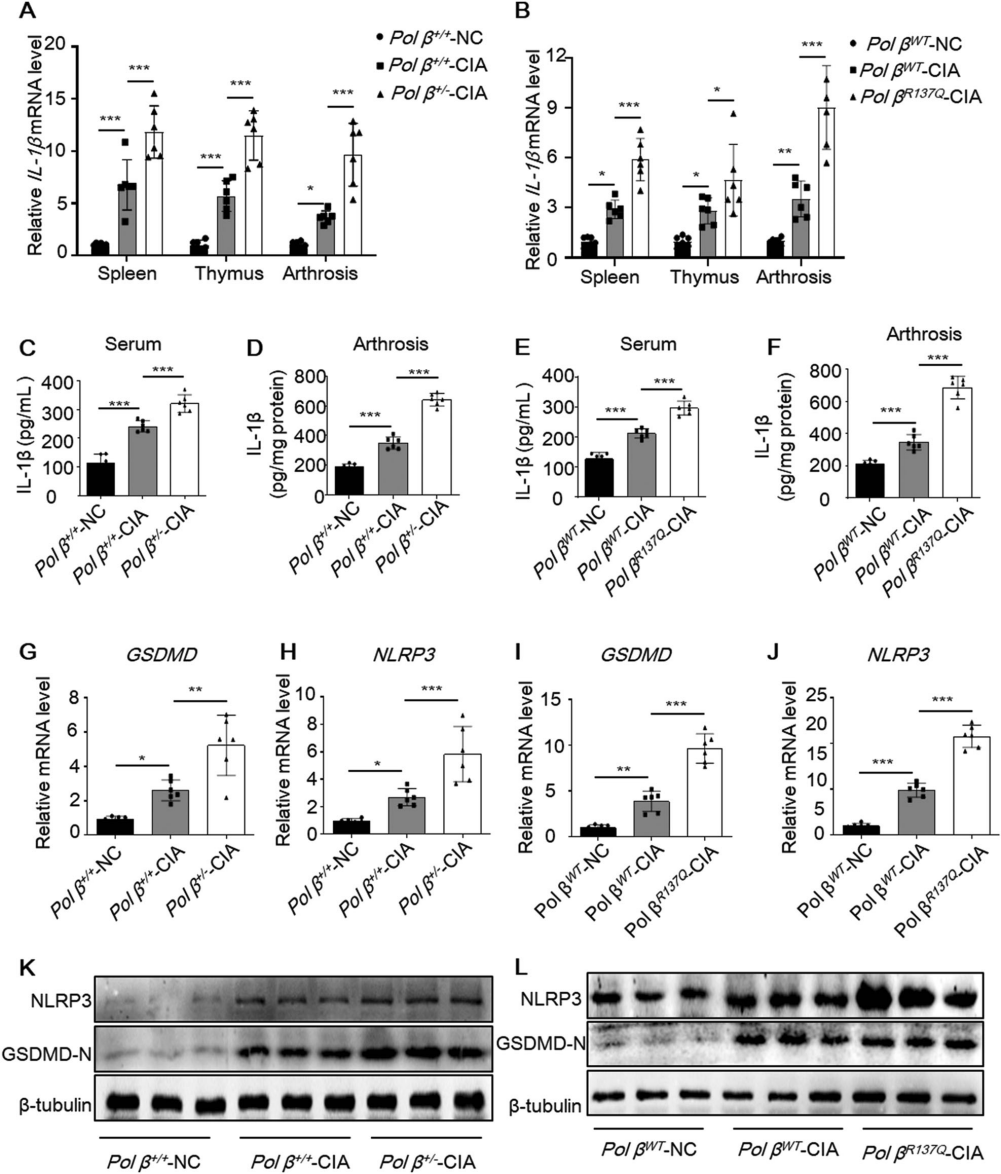
Pol β deficiency exacerbates the inflammatory response and pyroptosis in CIA mice. **A**, **B** qRT-PCR analysis of IL-1β mRNA expression in the spleen, thymus, and arthrosis of control and CIA mice. Data points indicate individual mice. Data present mean ± SD from at least 6 mice. *P < 0.05, **P < 0.01, ***P < 0.001, two-way ANOVA. **C**, **D** ELISA quantitation of IL-1β protein in the serum and arthrosis of *Pol β* ^*+/+*^-NC, *Pol β* ^*+/+*^-CIA and *Pol β* ^*+/−*^*-*CIA mice. **E**, **F** ELISA quantitation of IL-1β protein in the serum and arthrosis of *Pol β* ^*WT*^-NC, *Pol β* ^*WT*^-CIA, and *Pol β* ^*R137Q*^-CIA mice. **G**–**J** qRT-PCR analysis of GSDMD and NLRP3 mRNA expression in the ankles of control and CIA mice. Data points indicate individual mice, data present mean ± SD from at least 6 mice. *P < 0.05, **P < 0.01, ***P < 0.001, one-way ANOVA (**G**–**J**). **K**, **L** Western blot of NLR*P*3 and GSDMD-N in the ankles of control and CIA mice. β-tubulin was used as an internal control.

It has been established that macrophage pyroptosis plays an important role in RA progression, which can trigger the release of IL-1β and IL-18 and induce an uncontrollable inflammatory response [21]. The mRNA and protein levels of pyroptosis-related molecules such as NLRP3, caspase-1, IL-1β, and IL-18 in the PBMCs of RA patients were significantly higher than those of healthy subjects [7]. To investigate whether Pol β deficiency affects the expression of inflammatory factors, the mRNA levels of IL-1β and IL-18 in CIA mice were measured by qRT-PCR. Our data showed that the mRNA levels of both genes were significantly higher in the spleen, thymus, and arthrosis of *Pol β*^*+/−*^-CIA mice (Figs. 2A and S5A–C) and *Pol β*^*R137Q*^-CIA mice (Figs. 2B and S5D–F) than in *WT* mice. Accordingly, the ELISA data further confirmed the elevated protein expression of IL-1β in the serum and arthrosis of *Pol β*^*+/−*^-CIA mice (Figs. 2C, D and S5G) and *Pol β* ^*R137Q*^-CIA mice (Figs. 2E, F and S5H). These data revealed that Pol β deficiency exacerbated the expression of inflammatory factors. In addition, we examined the expression of NLRP3 and GSDMD-N to detect macrophage pyroptosis. The qRT-PCR and Western blotting results showed that NLRP3 and GSDMD-N were upregulated in *Pol β*^*+/−*^-CIA mice and *Pol β* ^*R137Q*^-CIA mice compared with *WT* mice (Fig. 2G–L).

**Fig. 3:**
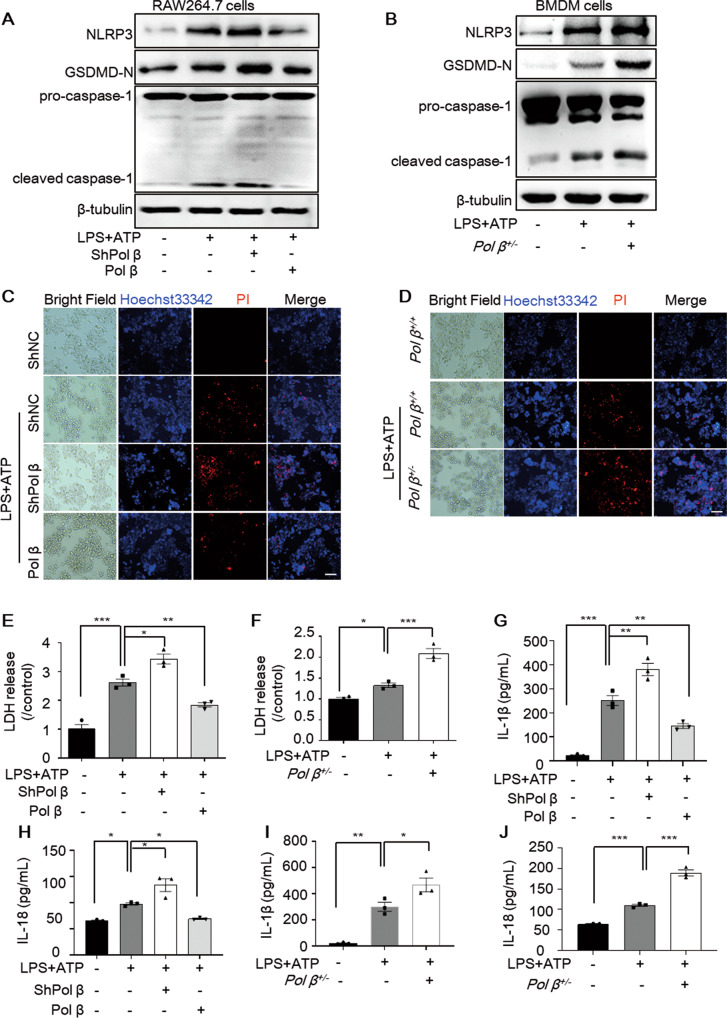
Pol β regulates pyroptosis in macrophages. **A**, **B** RAW264.7 cells with stable knockdown or overexpression of Pol β or BMDMs from *Pol β*^*+/−*^ mice were treated with 1 μg/mL LPS for 4 h, and 5 mM ATP was then added and incubated for an additional 1 h, and the levels of NLRP3, GSDMD-N, and caspase-1 expression were determined by Western blotting. **C**, **D** Representative images of Hoechst 33342 and PI double fluorescent staining. Scale bar, 50 μm. **E**, **F** LDH release levels in RAW264.7 cells after pyroptosis induction. Data present mean ± SEM of three independent experiments. *P < 0.05, **P < 0.01, ***P < 0.001, one-way ANOVA. ELISA analysis of IL**-**1β and IL-18 secreted by RAW264.7 cells with stable knockdown or overexpression of Pol β (**G**, **H**) or BMDMs from *Pol β*^*+/−*^ mice (**I**, **J**) after LPS plus ATP treatment. Data present mean ± SEM of three independent experiments *P < 0.05, **P < 0.01, ***P < 0.001, one-way ANOVA.

To further clarify whether Pol β affects macrophage pyroptosis, we first established stable RAW264.7 cell lines, which were infected with lentivirus expressing Pol β or Pol β shRNA (Fig. S6). We also isolated and cultured primary mouse bone marrow-derived macrophages (BMDMs) from *Pol β*^*+/−*^ transgenic mice or *Pol β*^*+/+*^ mice, which were identified with an F4/80-specific antibody (Fig. S7). These cells were treated with LPS plus ATP to induce pyroptosis (Fig. S8). Then, the expression of the pyroptosis-related molecules NLRP3, GSDMD-N, and cleaved caspase-1 was measured by Western blotting, and the results showed that the protein levels of these molecules were dramatically increased in Pol β-knockdown RAW264.7 cells or BMDMs from *Pol β*^*+/−*^ transgenic mice, while decreased in Pol β-overexpression cells (Fig. 3A, B). It has been reported that GSDMD can form membrane pores after being cleaved by activated caspase-1 during pyroptosis [22, 23]. To verify the role of Pol β in pyroptosis, the integrity of the cell membrane, a typical feature of pyroptosis, was examined by Hoechst 33342/PI double fluorescent staining. As shown in Fig. 3C, D, compared with the control group, Pol β-knockdown cells and BMDMs from *Pol β*^*+/−*^ transgenic mice exhibited membrane rupture, while this was rescued by Pol β overexpression. LDH release also showed the same effects in these cells (Fig. 3E, F). Moreover, we performed ELISA to measure the levels of IL-1β and IL-18 in the cell culture supernatant. Our data showed that after treatment with LPS plus ATP, more IL-1β and IL-18 were secreted in Pol β-deficient RAW264.7 cells or BMDM cells, but less in Pol β-overexpression cells (Fig. 3G–J). Taken together, these data indicated that Pol β regulated the inflammatory response and pyroptosis.

### Pol β deficiency exacerbates DNA damage and increases the leakage of dsDNA into the cytoplasm in pyroptotic macrophages

**Fig. 4:**
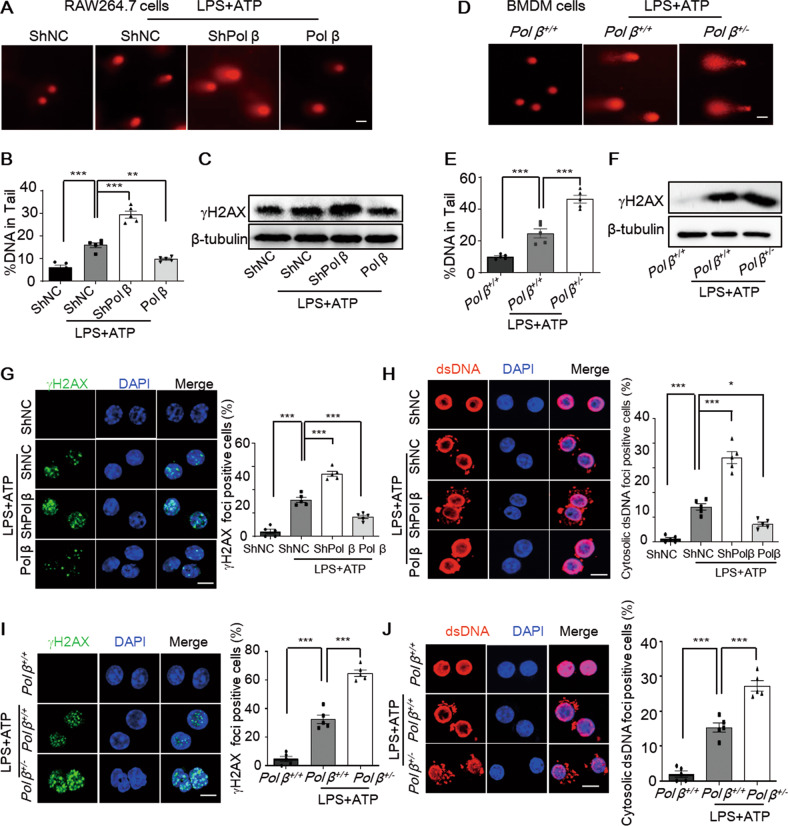
Pol β suppresses knockdown exacerbates DNA damage and decreases the leakage of dsDNA into the cytoplasm in pyroptotic macrophages. **A** Representative alkaline comet images of DNA damage in RAW264.7 cells after LPS and ATP treatment. **B** Percentage of DNA in the tails was quantified in panel A. Data represent mean ± SEM of three independent experiments. **P < 0.01, ***P < 0.001, one-way ANOVA. **C** Representative Western blot of **γ**-H2AX in RAW264.7 cells with stable knockdown or overexpression of Pol β after LPS plus ATP treatment. **D** Representative alkaline comet images of DNA damage in BMDM cells from WT and *Pol β*^*+/−*^ mice after LPS and ATP treatment. **E** Percentage of DNA in the tails was quantified in panel **D**. Data represent mean ± SEM of three independent experiments. ***P < 0.001, one-way ANOVA. **F** Representative Western blot of γ-H2AX in BMDM cells after LPS plus ATP treatment. Representative immunofluorescence images of γ-H2AX and cytosolic dsDNA foci in RAW264.7 cells (**G**, **H**) and BMDM cells (**I**, **J**) after treatment with LPS plus ATP. Lower panel: statistical analysis, data represent the mean ± SEM of three independent experiments; *P < 0.05, ***P < 0.001, one-way ANOVA. Scale bar, 10 μm.

Pol β is the key enzyme in base excision repair, and its deficiency may lead to the accumulation of DNA damage in cells. To explore the potential mechanism of Pol β in macrophage pyroptosis, we first used the alkaline comet assay to measure DNA damage in RAW264.7 cells treated with LPS and ATP. Our data showed that LPS plus ATP treatment significantly increased single- and double-strand DNA breaks in both cell lines. Moreover, as expected, Pol β deficiency exacerbated this effect that was rescued by Pol β overexpression (Fig. 4A, B). Next, Western blotting analysis of γ-H2AX, a well-characterized marker of DNA damage [24] further confirmed the results (Fig. 4C). These effects were also observed in BMDM cells (Fig. 4D–F). Additionally, similar results were obtained by immunofluorescence staining to detect γ-H2AX foci formation in RAW264.7 cells (Fig. 4G). The occurrence of double-stranded DNA (dsDNA) in the cytosol is a potent trigger of the innate immune system [25]. To investigate whether cytosolic dsDNA is involved in macrophage pyroptosis exacerbated by Pol β deficiency, immunofluorescence staining using an anti-dsDNA antibody was performed to visualize cytosolic dsDNA. The fluorescence signal revealed that the level of cytosolic dsDNA was significantly increased in all pyroptotic RAW264.7 cells induced by LPS plus ATP. Consistent with the DNA damage results, this increase was exacerbated by Pol β deficiency while rescued by Pol β overexpression (Fig. 4H). The formation of γ-H2AX foci and the level of cytosolic dsDNA were also significantly increased in BMDM cells from *Pol β*^*+/−*^ transgenic mice induced by LPS plus ATP (Fig. 4I–J). Taken together, our data showed that DNA damage was increased during macrophage pyroptosis and that Pol β deficiency exacerbated DNA damage and cytosolic dsDNA accumulation.

### The innate immune cGAS-STING pathway is activated by Pol β deficiency

It is well known that the cGAS-STING pathway can be aberrantly activated in autoimmune and inflammatory diseases and can also be activated by DNA damage [25]. Therefore, the exacerbation of the inflammatory response induced by Pol β deficiency may be due to the activation of the cGAS-STING pathway through the accumulation of DNA damage. To test this hypothesis and gain insight into the detailed mechanism by which Pol β regulates the inflammatory response, Pol β-knockout MEFs were used as the Pol β deficiency model in vitro, and knockout efficiency was confirmed by Western blotting (Fig. S9A). We first analyzed the levels of γ-H2AX, 53BP1, chromosome breakage, and chromosome aneuploidy to evaluate the effect of Pol β deficiency on DNA damage. We found that the levels of γ-H2AX (Fig. S9A) and 53BP1 (Fig. S9B, C) were increased in *Pol β*^*−/−*^ MEFs. Karyotype analysis indicated severe chromosome breakage and chromosome aneuploidy in *Pol β*^*−*/*−*^ MEFs (Fig. S9D). These results showed that Pol β deficiency increased DNA damage and chromosome breakage. Then, we measured dsDNA in the cytoplasm and found that the levels of cytosolic dsDNA were significantly increased in *Pol β*^*−/−*^ MEFs (Fig. S9E, F).

**Fig. 5:**
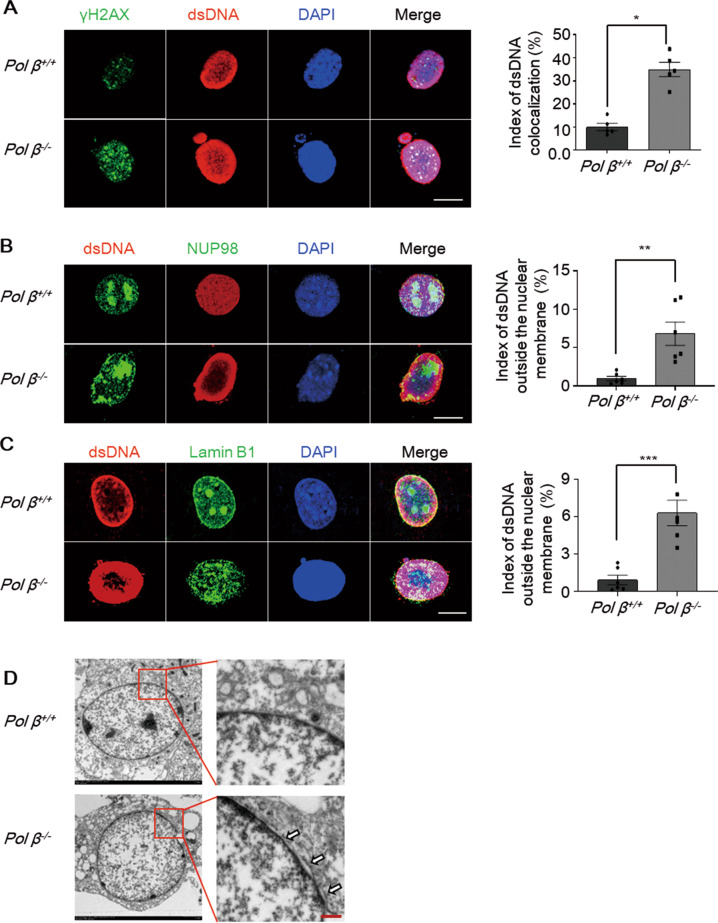
Export of damaged DNA from the nucleus to the cytosol. Representative confocal immunofluorescence images of dsDNA (red) colocalization with γ-H2AX (green) (**A**), NUP-98 (green) (**B**), and Lamin B1 (green) (**C**) in Pol β-knockout MEFs. Colocalization was visualized by using the Image J colocalization finder plugin. Colocalization in cells appears yellow in merged images. Right panel: quantification of the colocalization was performed by using the colocalization function of Image J from 100 cells. Data present mean ± SEM of three independent experiments. Scale bar, 10 μm. *P < 0.05, **P < 0.01, ***P < 0.001, Student’s *t*-test. **D** Abnormal nucleus membrane morphology in Pol β-knockout MEFs was observed by transmission electron microscopy. The White arrowheads indicate the expansion of intramembrous space between inner and outer nuclear membrane. Scale bar, 0.5 μm.

To investigate whether the cytosolic dsDNA was derived from nuclear DNA damage, we used immunofluorescence staining to examine the colocalization of γ-H2AX (Fig. 5A, green) and cytosolic dsDNA (Fig. 5A, red). In the Figure, yellow fluorescence indicates the colocalization of dsDNA and γ-H2AX. In *Pol β*^−/−^ MEFs, the overlap was significantly increased compared to that in WT MEFs (Fig. 5A).

Next, to further explore whether dsDNA leaked from the nucleus through the nuclear membrane, cells were immunostained with anti-nucleoporin 98 (NUP98) and anti-Lamin B1, which are two nuclear membrane envelope markers. Confocal fluorescence imaging revealed the presence of DNA buds and speckles close to the nuclear membrane, which indicated that nuclear membrane integrity might have been disrupted (Fig. 5B, C). Furthermore, we performed electron microscopy to examine nuclear membrane integrity and found that there were cracks between the internal and external nuclear membranes, and the nuclear membranes were partially fractured in *Pol β*^−/−^ MEFs (Fig. 5D). Collectively, our data suggested that Pol β deficiency increased DNA damage leaking to the cytosol via a compromised nuclear membrane.

**Fig. 6:**
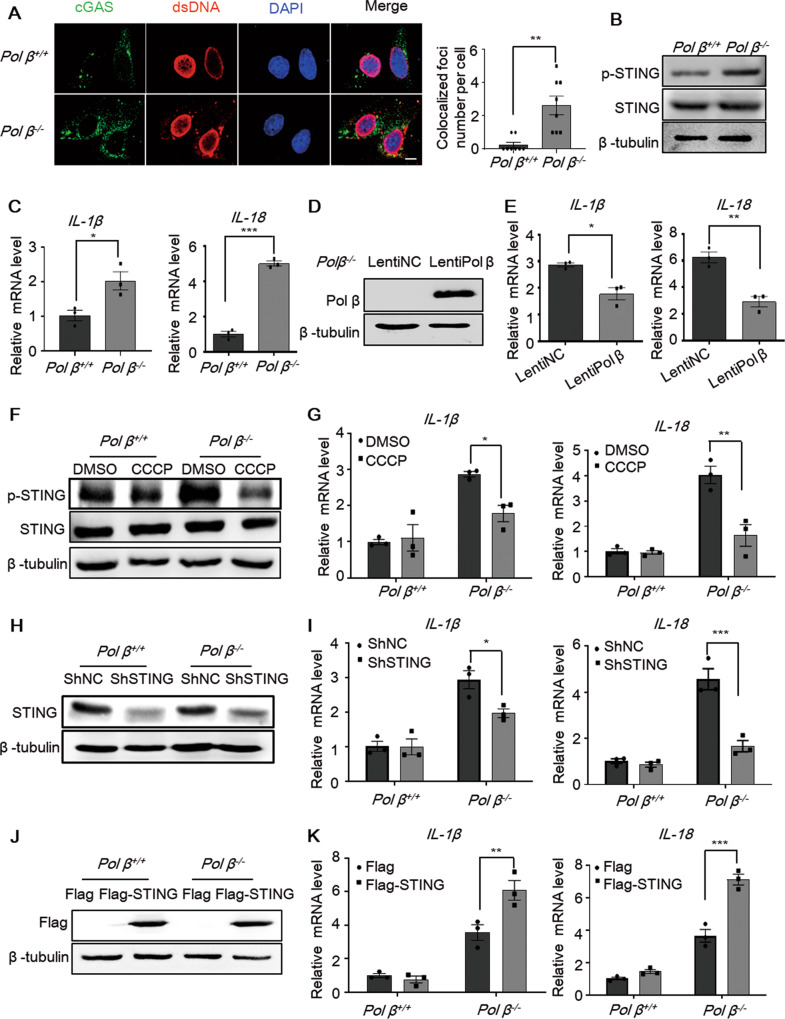
The cGAS-STING pathway is activated in Pol β-deficient cells. **A** Representative immunofluorescence images of the colocalization of cGAS (green) and dsDNA (red) in *Pol β* -knockout MEFs. Right panel: quantification of the colocalization was performed by using Image J from 100 cells. Data present mean ± SEM of three independent experiments, **P < 0.01, Student’s *t*-test. Scale bar, 10 μm. **B** Representative Western blot of STING phosphorylation in *Pol β*-knockout MEFs. **C** The mRNA levels of IL-1β and IL-18 were examined by qRT-PCR. Data present mean ± SEM of three independent experiments, *P < 0.05, ***P < 0.001, Student’s *t*-test. **D** Representative Western blot of Pol β overexpression in *Pol β*^*−/−*^ MEFs. **E** IL-1β and IL-18 mRNA levels test by qRT-PCR. Data present mean ± SEM of three independent experiments *P < 0.05, **P < 0.01, Student’s *t*-test. **F**–**J** The protein levels in wild-type and *Pol β*-knockout MEFs were examined after CCCP treatment (10 μM, 2 h) (**F**), STING knockdown (**H**), or STING overexpression (**J**). The relative mRNA levels of IL-1β and IL-18 in *Pol β*-knockout MEFs after CCCP treatment (**G**), STING knockdown (**I**) or STING overexpression (**K**). Data present mean ± SEM of three independent experiments. *P < 0.05, **P < 0.01, ***P  < 0.001 two-way ANOVA.

Once dsDNA leaks to the cytoplasm, the cytosolic DNA sensor cGAS can recognize cytosolic dsDNA and activate its downstream pathway. The colocalization of cGAS and dsDNA was examined by immunofluorescence analysis and suggested cGAS activation (Fig. 6A). The Western blot results showed that *Pol β*^−/−^ MEFs exhibited higher levels of phosphorylated STING than WT MEFs (Fig. 6B). In addition, IL-1β and IL-18 expression in *Pol β*^−*/*−^ MEFs was much higher than that in WT cells (Fig. 6C), while Pol β overexpression in *Pol β*^–^^/–^ MEFs partially weakened the induction of IL-1β and IL-18 (Fig. 6D, E). To examine the role of the cGAS-STING pathway in IL-1β and IL-18 expression induced by Pol β deficiency, we then treated the cells with CCCP, an inhibitor of STING phosphorylation (Fig. 6F). The real-time qPCR data showed that IL-1β and IL-18 induction in *Pol β*^−^^/−^ MEFs was decreased substantially with CCCP treatment (Fig. 6G). Moreover, STING knockdown by shRNA produced consistent results (Fig. 6H, I). The overexpression of STING increased the induction of IL-1β and IL-18 in *Pol β*^−*/*−^ MEFs (Fig. 6J, K). These data show that the cGAS-STING pathway plays an important role in the increases in IL-1β and IL-18 induced by Pol β deficiency.

### Pol β deficiency exacerbates pyroptosis in macrophages via the cGAS-STING-NF-κB pathway

**Fig. 7:**
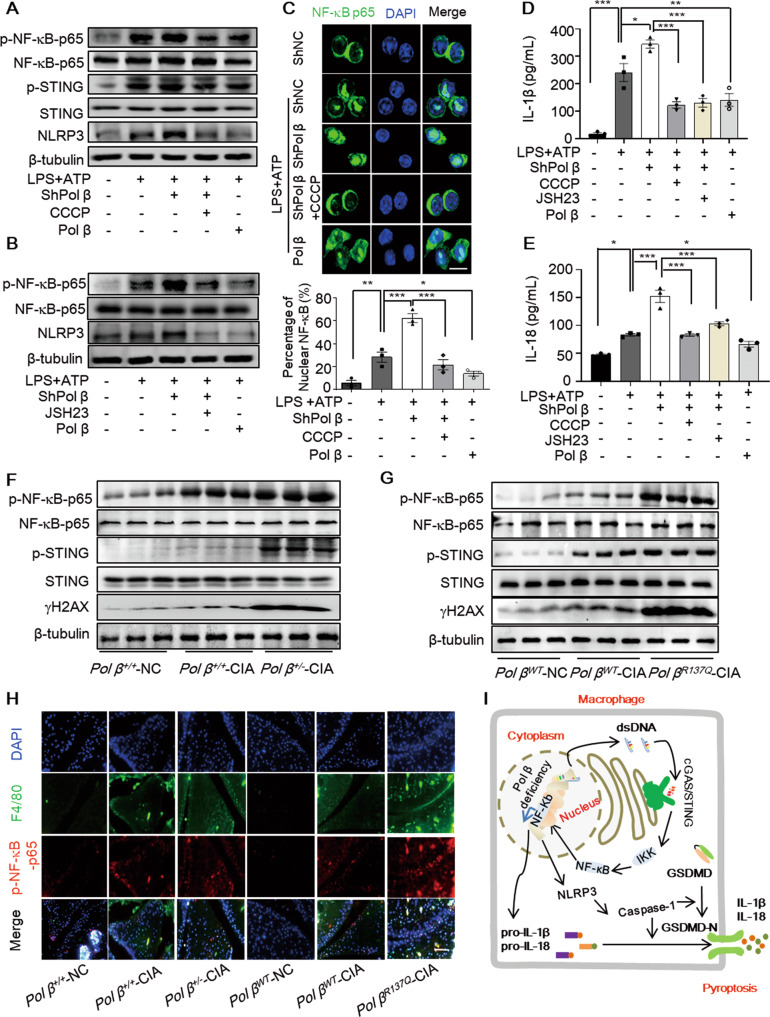
Pol β deficiency exacerbates macrophages pyroptosis via the cGAS/STING/NF-κB pathway in vitro and in vivo. **A**, **B** RAW264.7 cells were pretreated with 10 μM CCCP or 10 μM JSH-23 for 2 h, treated with 1 μg/mL LPS for 4 h, and then treated with 5 mM ATP for the final hour. The protein levels were examined by Western blotting. Data are representative blot of three independent experiments. **C** Representative images of NF-κB p65 staining. Scale bar, 10 μm. Lower panel: statistical analysis, the data represent the mean ± SEM from more than 100 cells for each group; *P < 0.05, **P < 0.01, ***P < 0.001, one-way ANOVA. The levels of IL-1β (**D**) and IL-18 (**E**) secreted by macrophages were analyzed by ELISA. Data present the mean ± SEM of three independent experiments, *P < 0.05, **P < 0.01, ***P < 0.001, one-way ANOVA. The protein levels in the ankles of Pol β knockdown-CIA mice (**F**) and Pol β R137Q-CIA mice (**G**). Data are representative blot of three independent experiments. **H** Representative immunofluorescence images showing NF-κB p65 phosphorylation in macrophages in the ankles of CIA mice. Scale bar, 500 μm. **J** Schematic diagram showing our proposed mechanisms of Pol β deficiency exacerbating macrophage pyroptosis through activating the cGAS-STING-NF-κB pathway.

DNA is a potent activator of inflammatory responses, and among a dozen DNA sensors, the cGAS-STING pathways are central to nucleic acid sensing [26]. cGAS–STING signaling can lead to the activation of NF-κB and the induction of inflammatory cytokines [27, 28]. After confirming the effect of Pol β on macrophage pyroptosis, we next investigated whether the cGAS-STING-NF-κB pathway was involved in the molecular mechanisms of this effect. As shown in Fig. 7A, B, p-STING and p-NF-κB-p65 were increased in RAW264.7 cells treated with LPS and ATP, and these levels were further increased by Pol β knockdown and decreased by Pol β. However, pretreatment with CCCP effectively abolished the activation of STING and NF-κB p65 induced by Pol β knockdown. Subsequently, the upregulation of NLRP3 was also abolished by CCCP or JSH-23 (an NF-κB-p65 phosphorylation inhibitor) pretreatment in Pol β-knockdown macrophages (Fig. 7A, B). Moreover, Pol β deficiency in BMDMs induced the same effects (Fig. S10A, B).

Next, we investigated whether Pol β regulates the nuclear translocation of NF-κB-p65. Immunofluorescence analysis showed that NF-κB-p65 nuclear translocation was increased in pyroptotic macrophages and Pol β knockdown enhanced this effect, which was inhibited by CCCP pretreatment or Pol β overexpression (Figs. 7C and S10C). In addition, ELISA was performed to measure the secretion of IL-1β and IL-18, and the results confirmed that the Pol β knockdown-induced elevation in IL-1β and IL-18 was also abolished by CCCP or JSH-23 treatment (Figs. 7D, E and S10D, E). Taken together, these results indicated that Pol β regulated pyroptosis in macrophages via the cGAS-STING-NF-κB pathway.

We further investigated whether the molecular mechanism by which Pol β deficiency exacerbates inflammation is related to the cGAS-STING-NF-κB pathway in CIA mice. The results showed that the levels of p-STING, p-NF-κB-p65, and γ-H2AX were notably upregulated in the ankles of CIA mice, and these levels could be further upregulated by Pol β deficiency (Fig. 7F, G). Additionally, it was observed that the levels of p-NF-κB-p65 in the ankles of *Pol β*^*+/−*^-CIA or *Pol β*^*R137Q*^-CIA mice were markedly increased compared to those in *WT* mice, as shown by immunofluorescence assays (Fig. 7H). Overall, our results provide clear evidence that Pol β is involved in CIA through the cGAS-STING-NF-κB pathway in vivo.

## Discussion

In the present study, we demonstrate that Pol β plays a role in RA pathogenesis and that its deficiency exacerbates disease severity in CIA mice. Pol β deficiency exacerbates DNA damage and increases the leakage of dsDNA into the cytoplasm, thereby activating the cGAS-STING-NF κB pathway and exacerbating the inflammatory response and macrophage pyroptosis (Fig. 7I).

Pol β is a key BER enzyme that maintains genomic integrity and stability, and Pol β knockout in mice causes early embryonic lethality. Several lines of evidence have shown that Pol β mutations are associated with inflammation and innate immunity [15, 16]; however, the detailed mechanism is still not fully understood. Sweasy et al. revealed that mice carrying the Y265C Pol β mutant developed several SLE-associated pathologies, which was due to aberrant V(D)J recombination and a high frequency of somatic hypermutation (SHM) [15]. Consistent with their findings, our data showed that Pol β was involved in inflammation. Although no obvious abnormalities were observed in Pol β transgenic mice, however, the inflammatory factors detected were significantly upregulated. Moreover, Pol β deficiency exacerbated the inflammatory response and disease severity in CIA mice. This might be owing to the fact that Pol β functions under DNA damage stress. Our previous study showed that the Pol β mutant Pol β^R137Q^ possesses lower polymerase activity and an unpaired ability to interact with proliferating cell nuclear antigen (PCNA), resulting in deficient DNA repair capacity in reconstitution assays and reduced BER efficiency [19, 20]. Therefore, in this study, Pol β deficiency increased DNA damage in macrophages and MEFs. Moreover, our results suggest that the gradual accumulation of DNA lesions leads to cytosolic DNA leakage because there was increased colocalization of cytosolic dsDNA with γ-H2AX (a typical DNA double strand break marker) and NUP98/Lamin B1 (two nuclear membrane envelope markers). Indeed, in addition to Pol β, deficiencies in other DNA damage sensors or DNA repair factors can also lead to the accumulation of DNA damage and nuclear cytosolic DNA leakage [29].

The accumulation of unrepaired DNA lesions in the cytoplasm will activate the innate immune cGAS-STING pathway; subsequently, STING activates the transcription factors IRF3 and NF-κB via the kinases TBK1 and IKK, respectively. IRF3 and NF-κB translocate into the nucleus to induce the expression of IFNs and other cytokines [30–33]. It has also been reported that single-stranded DNA fragments are released from stalled forks and accumulate in the cytoplasm, where they activate the cGAS-STING pathway, causing a severe congenital inflammatory disease known as Aicardi–Goutières syndrome [34]. Taking these findings together, it appears that cells are capable of inducing many DNA-sensing pathways to recognize damaged self-DNA in intracellular compartments or in the cytoplasm. This chronic inflammation is believed to stem from persistent genomic instability driven by a lack of appropriate DNA repair. Consistent with these findings, we demonstrated that in Pol β^-^knockdown macrophages, the cGAS-STING pathway was activated by DNA damage and cytosolic leakage, and NF-κB p65 was then phosphorylated and translocated into the nucleus. Accordingly, high levels of IL-1β and IL-18 were induced in Pol β-knockdown macrophages. More importantly, *Pol β*^*+/−*^-CIA mice and *Pol β*^*R137Q*^-CIA mice exhibited significantly increased levels of p-STING, p-NF-κB p65, IL-1β, and IL-18 compared with WT mice. Collectively, our study provides a novel explanation of the role of Pol β in inflammation mediated by the cGAS/STING pathway.

In addition to cGAS, several proteins have been suggested to function as DNA sensors, including DDX41, interferon γ-inducible protein 16 (IFI16), absent in melanoma 2 (AIM2), LSm14A (also called RAP55), meiotic recombination 11 (MRE11), DNA-dependent protein kinase (DNA-PK), DNA-dependent activator of IRFs (DAI), and RNA polymerase III [29, 35–41]. Among these cytosolic dsDNA sensors, cGAS and AIM2 play important roles in macrophage pyroptosis, and both factors increase proinflammatory IL-1β and IL-18 expression via the activation of caspase-1. In this study, we focused on the cGAS/STING pathway because self-dsDNA is mainly recognized by cGAS, and AIM2 is crucial for protecting from DNA viruses and some cytosolic bacterial pathogens [42]. However, we cannot completely rule out the possibility that these sensors are relevant to STING activation and macrophage pyroptosis in the pathological mechanism of RA, and we will continue to study this in the future.

As a rapidly inducible transcription factor, NF-κB has a wide role in inflammation and other physiological or pathological processes, such as cell proliferation, metastasis, metabolic changes, and apoptosis [43]. Recent studies have focused on NF-κB in pyroptosis, especially macrophage pyroptosis. It was reported that NF-κB was necessary but not sufficient for NLRP3 activation, and a second stimulus, such as ATP or crystal-induced damage, was required for NLRP3 activation. In addition, cleaved caspase-1 and the NLRP3 inflammasome were dose-dependently reduced by a specific inhibitor of NF-κB [44]. Moreover, histone deacetylase 6 (HDAC6) and tannic acid can inhibit NLRP3 inflammasome-mediated IL-1β production by blocking NF-κB signaling in macrophages [45, 46]. Interestingly, A20 deficiency in macrophages significantly enhanced NLRP3 inflammasome-mediated caspase-1 activation, pyroptosis, and IL-1β secretion, and negative regulation of the NLRP3 inflammasome by A20 protected against rheumatoid arthritis [8]. Our data revealed that the activation of NF-κB p65 was essential for Pol β deficiency-mediated exacerbation of macrophage pyroptosis because the increases of NLRP3 and IL-1β expression were blocked by the NF-κB p65-specific inhibitor JSH-23. Additionally, the STING inhibitor CCCP had the same effect as JSH-23. Overall, Pol β deficiency exacerbated pyroptosis in macrophages via the cGAS/STING/NF-κB pathway.

It is well established that various kinds of immune cells, including macrophages, mast cells, natural killer cells, and neutrophils, are involved in rheumatoid arthritis [1]. Among them, macrophages play a key role in the pathogenesis of RA. The activation of macrophage pyroptosis leads to the release of proinflammatory mediators, including cytokines, alarmins, IL-1β, and IL-18, which in turn promote inflammation by recruiting additional immune cells, polarizing T cells, and activating fibroblasts [3]. Here, we established CIA models in *Pol β*^*+/−*^ and *Pol β*^*R137Q*^ transgenic mice, and the data showed that Pol β deficiency exacerbated disease severity in CIA mice. Moreover, our findings confirmed that the levels of IL-1β and IL-18 and the pyroptosis-related molecules NLRP3 and GSDMD-N in macrophages were significantly upregulated by Pol β deficiency. Collectively, our in vivo and in vitro data suggest that Pol β can affect RA via macrophage pyroptosis.

In conclusion, we revealed a novel role of Pol β in the autoimmune disease RA. DNA damage caused by Pol β deficiency can activate the cGAS/STING/NF-κB signaling pathway, upregulate the expression of NLRP3, IL-1β, and IL-18, exacerbate macrophage pyroptosis and regulate the occurrence and development of RA, while Pol β overexpression downregulates inflammatory cytokines and impair macrophage pyroptosis. Taken together, Pol β regulates RA by inhibiting macrophage pyroptosis via the cGAS/STING/NF-κB Pathway. These findings provide clear evidence of the role of Pol β in RA and suggest that Pol β may be an effective target for the prevention and treatment of RA and other relevant autoimmune diseases.

## Materials and methods

### Reagents and antibodies

DMEM, RPMI 1640, and Opti-MEM medium were purchased from Thermo Fisher Scientific (Waltham, MA, USA). Fetal bovine serum, Hoechst 33342, propidiumiodide (PI), penicillin G sodium salt, lipopolysaccharide (LPS), Freund’s complete adjuvant, and chicken type II collagen were obtained from Merck (Darmstadt, Germany). Antibodies against NLRP3, GSDMD, caspase-1, NF-κB, and p-NF-κB were obtained from Cell Signaling Technology (Danvers, MA, USA). Antibodies against Tubulin, STING, p-STING, F4/80, and γ-H2AX were obtained From Abcam (Cambridge, United Kingdom). Mouse IL-1β and IL-18 uncoated ELISA kits were purchased from Thermos Fisher Scientific (Waltham, MA, USA). Lactate dehydrogenase (LDH) assay kits were obtained from Nanjing Jiancheng Bioengineering Institute (Nanjing, China). TRIzol reagent, PrimeScript RT Reagent Kit with gDNA Eraser, Effectene Transfection Reagent, and SYBR Premix Ex TaqII were obtained from Vazyme (Nanjing, China). CCCP and JSH-23 were purchased from MCE (New Jersey, USA). Human or mouse PBMC separation medium were obtained from Solarbio (Beijing, China).

### Animals and collagen-induced arthritis (CIA) mouse model

Pol β^+/−^ mice on a C57BL/6 genetic background and their wild-type littermates were obtained from GemPharmatech Co., Ltd. (Nanjing, China). Pol β^R137Q^ mice and their wild-type (WT) littermate 129S1 mice were gifts from Professor Binghui Shen (Beckman Research Institute, City of Hope, Duarte, CA, USA).

6–8 weeks male mice were maintained on a 12 h light/dark cycle at 22–24 °C and 40–70% humidity and had free access to rodent chow and tap water. After adaptive feeding for 7 days, the CIA mouse model was induced according to previous studies [47]. Briefly, chicken type II collagen was emulsified in an equal volume of Freund’s complete adjuvant on ice. Then, the mice were immunized with a single subcutaneous injection around the base of the tail with 100 μL of the emulsion (100 μg of chicken type II collagen) containing 1 mg/mL *Mycobacterium tuberculosis*. Arthritic scores and body weight were determined by two independent, blinded observers twice per week. Scoring was performed with a 0–4 scale as follows: 0: normal; 1: mild swelling; 2: moderate swelling (or mild swelling + 1 or 2 swollen joints); 3: swelling of all joints; and 4: joint distortion and/or rigidity and dysfunction. Each hind limb was graded, and the maximum possible score was 8 for each mouse. A mouse with a score of one or above was regarded as arthritic. The animal experiment was performed in accordance with the Regulations of Experimental Animal Administrations issued by the Laboratory Animal Care Committee at Nanjing Normal University (Ethical code: IACUC-1903029).

### Cell culture and stable cell lines

RAW264.7 cells were obtained from the China Infrastructure of Cell Line Resources (Beijing, China) and cultured in complete medium consisting of DMEM supplemented with penicillin (100 U/mL), streptomycin (100 μg/mL) and 10% fetal bovine serum (FBS) in a humidified 5% CO_2_ atmosphere at 37 °C. To knockdown Pol β or overexpression Pol β, we used the lentivirus vector pGLV3 containing the shRNA sequence or mouse full-length Pol β. The following oligonucleotides were used: ShPol β: 5′-GGAGCTGAAGCTAAGAAA TTG-3′. Mouse full-length STING was cloned into pFlag-CMV4 plasmids. The mouse STING shRNA plasmid was constructed by our lab with the pSilencer 3.0 H1 plasmid with the sequence 5′-CAACATTCGAT TCCGAGATAT-3′. Wild-type and Pol β-knockout mouse embryonic fibroblasts (MEFs) were a gift from Professor Binghui Shen. Primary bone marrow-derived macrophages (BMDMs) were isolated from the tibia and femoral bone marrow cells of mice as previously described [48] and cultured in RPMI 1640 supplemented with 10% FBS, 1 mM sodium pyruvate, and 2 mM L-glutamine in the presence of 50 ng/mL granulocyte macrophage colony stimulating factor (GM-CSF). Cell cultures were replenished with fresh medium every 3 days, and BMDM purity was determined (>95%) on the seventh day by immunofluorescence staining with a monoclonal antibody against F4/80.

### Human PBMC isolation

PBMCs were isolated from the anticoagulant-treated blood of active RA patients (*n* = 8) and healthy donors (*n* = 7) according to the manufacturer’s instructions. This study was approved by the Ethics Committee of The Affiliated Drum Tower Hospital of Nanjing University Medical School (Ethical code: 202127901). All study subjects provided written informed consent.

### Induction of macrophage pyroptosis

LPS plus ATP was used to induce pyroptosis in RAW264.7 cells. To determine the optimal pyroptosis stress conditions, RAW264.7 cells were treated with 0–1 μg/mL LPS for 5 h, and the concentration of 1 μg/mL increased NLRP3 levels more than twofold. Then, RAW264.7 cells were treated with 1 μg/mL LPS for 4 h, and 5 mM ATP was added and incubated for an additional 0–1 h. Treatment with 5 mM ATP for 1 h dramatically affected NLRP3 levels. Accordingly, treatment with 1 μg/mL LPS for 4 hours and incubation with 5 mM ATP for an additional hour was selected for subsequent experiments.

### Western blot analysis

Cells or tissues were homogenized in cold RIPA lysis buffer contained PMSF, and the supernatant was harvested by centrifugation. After protein quantification using a bicinchoninic acid (BCA) Protein Assay Kit, 30 µg total proteins were separated by SDS-PAGE gel and transferred to 0.22 μm PVDF membrane. The membranes were blocked with blocking buffer (5% non-fat milk powder in TBST: 10 mM Tris-HCl pH 8.0, 150 mM NaCl, and 0.05% Tween 20) for 1 h at room temperature, and then were incubated with primary antibodies at 4 °C overnight, followed by incubation with appropriate horseradish peroxidase-conjugated secondary antibody for 1–2 h at room temperature. They were finally exposed to ECL western blot detection reagents.

### Immunofluorescence staining

Cells were fixed in 4% paraformaldehyde for 30 min and followed by incubation with 0.1% Triton-100 for 15 min at room temperature. Then cells were blocked with 5% BSA and incubated with the primary antibody at 4 °C overnight. After incubating with the secondary antibody, cells were stained with DAPI for 10 min. Subsequently, images were acquired by using Nikon 80I 10–1500X microscope. Negative control was performed by omitting the primary antibody and revealed no labeling (data not shown).

For histological analysis, tissues were fixed in 4% paraformaldehyde at 4 °C overnight, embedded in paraffin, and sliced. After deparaffinization and hydration, tissues were pretreated by sodium citrate solution for the antigen retrieval. Then blocking with 5% goat serum for 30 min at room temperature, tissue sections were incubated with primary antibody at 4 °C overnight, followed by incubating with the secondary antibody for 1 h at 37 °C in the dark. After counterstaining with DAPI to show cell nucleus, tissue sections were photographed by Nikon 80I 10-1500X microscope. Quantification of colocalization was analyzed by Image J colocalization finder plugin.

### Histopathological assessment

The joint was excised and fixed in 4% paraformaldehyde at 4 °C overnight. After decalcification with 10% EDTA, joint tissue was embedded in paraffin, cut into 5 μm-thick slices, and stained with hematoxylin-eosin. Images were obtained with a Nikon 80I 10-1500X microscope. Histopathological analysis was evaluated by independent observers in a blinded manner.

### Safranin O/fast green staining

The cartilage injury was identified by safranin O/fast green staining according to the manufacturer’s instructions. The paraffin-embedded slices were deparaffinized, sequentially were stained with hematoxylin for 5 min, and acid differentiated for 15 s. Then slices were stained with fast green for another 5 min, dried to discard excessive dying solution until the cartilage was colorless. For Safranin staining, the slices were stained with safranin staining solution for 5 min and dehydrated quickly with absolute ethanol. Finally, slices were mounted with neutral gum and photographed by microscope. Staining results: The cartilage matrix was dark red, the chondrocyte cytoplasm was red, the nucleus was gray black, and the bone tissue was gray green or green. Cartilage damage was performed with a 0–4 scale as follows [49]: 0: normal; 1: mild loss of Safranin O staining with no obvious chondrocyte loss; 2: moderate loss of staining with focal mild chondrocyte loss; 3: marked loss of staining with multifocal marked chondrocyte loss; and 4: severe diffuse loss of staining with multifocal severe chondrocyte loss.

### Enzyme-linked immunosorbent assay (ELISA)

The protein levels of IL-1β or IL-18 were measured by specific ELISA kits according to the manufacturer’s instructions. Briefly, 96-well ELISA plates were incubated with capture antibody (pretitrated, purified anti-mouse IL-1β or IL-18 antibody) overnight at 4 °C and then blocked with ELISA/ELISPOT Diluent at room temperature for 1 h. Samples (100 μL/well) or prepared standards were added to the appropriate wells and incubated overnight at 4 °C for maximum sensitivity. After being incubated with detection antibody (pretitrated, biotin-conjugated anti-mouse IL-1β or IL-18 antibody) at room temperature for 1 h, the samples were treated with streptavidin-HRP for 30 min. Finally, the samples were incubated with TMB substrate solution, and the absorbance was read at 450 nm using a microplate spectrophotometer.

### Alkaline Comet assay

Alkaline Comet assay was performed using a Comet assay kit according to the manufacturer’s recommendations. Briefly, after treatment, cells were harvested and mixed with low-melting-point agarose, subsequently plated on a comet assay glass slide. After lysis and electrophoresis (25 V, 30 min), cells were stained with propidium iodide (PI). Images were obtained by a fluorescence microscope. The percentage of DNA in tails was quantified by Comet assay software project (CASP).

### Reverse transcription-quantitative PCR (qRT-PCR) analysis

Total mRNA was isolated from cells and tissues by using TRIzol reagent according to the manufacturer’s instructions and quantified by using Thermo NanoDrop 2000. Then, 1 μg total RNA was reverse transcribed into cDNA. qRT-PCR was performed using a reaction mixture with SYBR according to the manufacturer’s protocols. All PCR amplifications were performed in triplicate and repeated in three independent experiments. Primers for each gene were showed in Table S1.

### Hoechst 33342/PI double staining

Morphological analysis was observed by Hoechst 33342/PI double staining. Cells were cultured in six-well plates at a density of 2 × 10^5^ cells per well. After treatment, cells were stained with Hoechst 33342 (10 μg/ml) for 30 min at room temperature, followed with PI (1 μg/ml) for 5 min in the dark. Cells were photographed by using a fluorescence microscope.

### LDH release assay

When the cell membrane is destroyed by pyroptosis, enzymes in the cytoplasm are released into the culture medium, including the relatively stable LDH. By measuring the activity of LDH released into the culture medium by membrane-ruptured cells, the integrity of the cell membrane can be examined. LDH in the culture medium was measured by using commercial kits in 96-well enzyme immunoassay plates according to the manufacturer’s instructions.

### Karyotype analysis

Chromosome breakage and chromosome aneuploidy were detected by karyotype analysis. Cells were collected and treated with colcemid to arrest cells at metaphase, then incubated with hypotonic solution (75 mM KCl) for 20 min at room temperature. Subsequently, cells were fixed with Carnoy’s solution three times and stained with Giemsa solution. Finally, the images were recorded and analyzed with ImagePro 7.0 (Media Cybernetics, Bethesda, MD, USA), and the chromosomes in each metaphase cell were counted.

### Transmission electron microscopy

The collected cells were fixed with 2.5% glutaraldehyde at 4 °C overnight and washed three times. Then, the cells were postfixed in 1% osmic acid at 4 °C for 2 h and dehydrated with a series of ethanol solutions. After being embedded in Spurr’s epoxy resin, the samples were cut into 50–60 nm ultrathin sections. Finally, the images were photographed by a JEOL JEM-100CX electron microscope.

### Statistical analysis

Statistical analysis was performed by one-way ANOVA, two-way ANOVA or Student’s *t*-test by using Prism 8.0 software (GraphPad Software, La Jolla, CA, USA). Values of *P* less than 0.05 were considered statistically significant.

## Supplementary information

supplementary material

original western blots

aj-checklist

author list confirmation

## Data Availability

All data are available in the main text or the supporting information.
