# Supplementary material for: A novel mechanism for macrophage pyroptosis in rheumatoid arthritis induced by Pol β deficiency
Source: Cell Death Dis. 2022 Jul 6;13(7):583. doi: 10.1038/s41419-022-05047-6 (PMC9259649; doi:10.1038/s41419-022-05047-6)
Supplement: Supplementary file 1 — supplementary material [file 41419_2022_5047_MOESM1_ESM.docx]

**A novel mechanism for macrophage pyroptosis in rheumatoid arthritis induced by Pol β deficiency**

Lili Gu^1a^, Yuling Sun^1a^, Ting Wu^1^, Ge Chen^1^, Xiaojun Tang^2^, Lianfeng Zhao^1^, Lingfeng He^1^, Zhigang Hu^1^, Lingyun Sun^2^, Feiyan Pan^1^*, Zhimin Yin^1^*, Zhigang Guo^1^*

^1^Jiangsu Key Laboratory for Molecular and Medical Biotechnology, College of Life Sciences, Nanjing Normal University, 1 Wen Yuan Road, Nanjing, China 210023.

^2^Department of Rheumatology and Immunology, The Affiliated Drum Tower Hospital of Nanjing University Medical School, Nanjing, China 210008

**Supplementary Information**

The following file contains supplementary material for the paper “DNA polymerase β deficiency exacerbates rheumatoid arthritis by exacerbating macrophage pyroptosis via the cGAS-STING-NF-κB pathway”.

This file is composed of:

- Supplementary tables (2 tables)
- Supplementary figures and relative supplementary figure legends (10 figures)

**Supplementary Table 1. Primer sequences used for qRT-PCR**

| Gene | Primer sequence (5ʹ → 3ʹ) |
| --- | --- |
| Homo-β-actin | Sense: CTCTTCCAGCCTTCCTTCCT  Antisense: CAGGGCAGTGATCTCCTTCT |
| Homo-Pol β | Sense: CATGGGTGTTTGCCAGCTTC  Antisense: AGGGACGGATGGTGTACTCA |
| Mus-Pol β | Sense: CGTGCTGGAAAGGCAAATCT  Antisense: CCTTCAGAAAGACTGCCAGC |
| Mus-β-actin | Sense: GAAATCGTGCGTGACATCAAAGAG  Antisense: CAATAGTGATGACCTGGCCGTC |
| Mus-NLRP3 | Sense: ATTACCCGCCCGAGAAAGG  Antisense: CATGAGTGTGGCTAGATCCAAG |
| Mus-GSDMD | Sense: CCATCGGCCTTTGAGAAAGTG  Antisense: ACACATGAATAACGGGGTTTCC |
| Mus-IL-1β | Sense: AGCTTCAGGCAGGCAGTATC  Antisense: AGTCACAGAGGATGGGCTCT |
| Mus-IL-18 | Sense: AGTGAACCCCAGACCAGACT  Antisense: TGGCAAGCAAGAAAGTGTCC |
| Mus-IFNα | Sense: GCAATCCTCCTAGACTCACTTCTGCA  Antisense: TATAGTTCCTCACAGCCAGCAG |
| Mus-IFNβ | Sense: AGCTGAAGCAGTTCCAGAAG  Antisense: AGTCTCATTCCAGCCAGTGC |

| No. | Gender | Age | Disease duration  ( months) | RF  (IU/mL) | CRP  (mg/L) | Anti-CCP  ( RU/ml) | ESR  (mm/h) | DAS28 | VAS score | Morning stiffness  duration (hours) | SJC | TJC |
| --- | --- | --- | --- | --- | --- | --- | --- | --- | --- | --- | --- | --- |
| 1 | F | 68 | 2 | 77.7 | 23.2 | 195.6 | 60 | 6.4 | 6 | 1.0 | 8 | 10 |
| 2 | F | 56 | 6 | 67.5 | 4.7 | 147.3 | 18 | 5.1 | 5 | 3.5 | 7 | 8 |
| 3 | M | 49 | 13 | 20.3 | 18.9 | 4.5 | 14 | 5.2 | 5 | 1.0 | 9 | 20 |
| 4 | F | 53 | 35 | 189.3 | 46.9 | 3.5 | 34 | 6.2 | 6 | 1.5 | 10 | 16 |
| 5 | F | 63 | 56 | 89.6 | 5.1 | 74.1 | 70 | 5.4 | 3 | 0.5 | 4 | 4 |
| 6 | F | 73 | 11 | 229．4 | 5.0 | 200.8 | 43 | 6.5 | 7 | 4.0 | 11 | 18 |
| 7 | F | 61 | 7 | 6.6 | 31.1 | 317.7 | 60 | 4.8 | 3 | 1.0 | 5 | 8 |
| 8 | M | 67 | 34 | 26.1 | 7.8 | 87.6 | 20 | 4.7 | 4 | 1.5 | 7 | 10 |

**Table S2 Basic information of RA patients**

**Supplementary Figures**


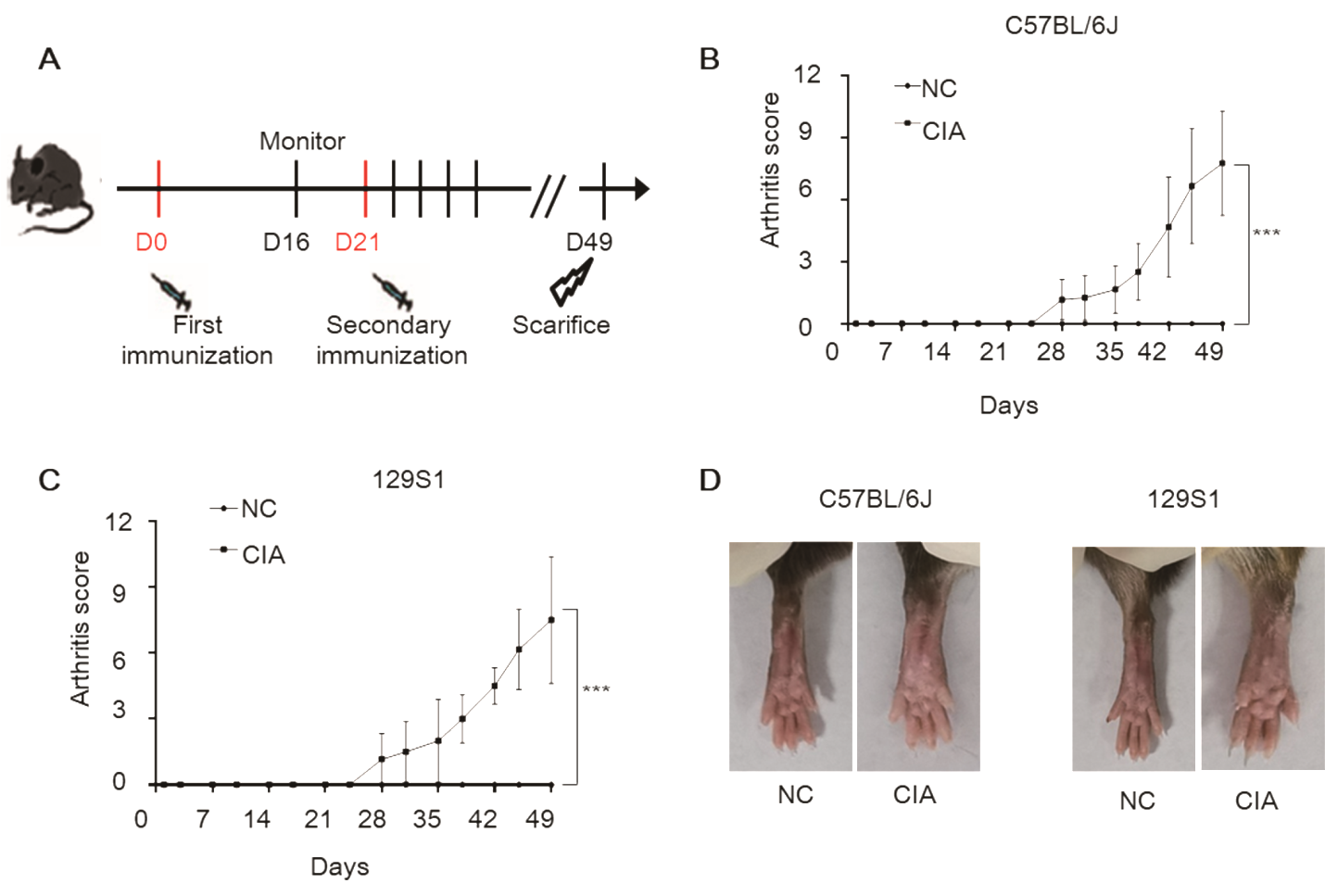


**Fig. S1. Construction of a collagen-induced arthritis (CIA) mouse model. (A)** Schematic illustrating the establishment of the CIA mouse model. **(B, C)** Clinical arthritic scores of CIA mice. Data are means ± SD from at least 6 mice. ^***^*P*<0.001, two-way ANOVA. **(D)** Representative macroscopic images of CIA mice from at least 6 mice.


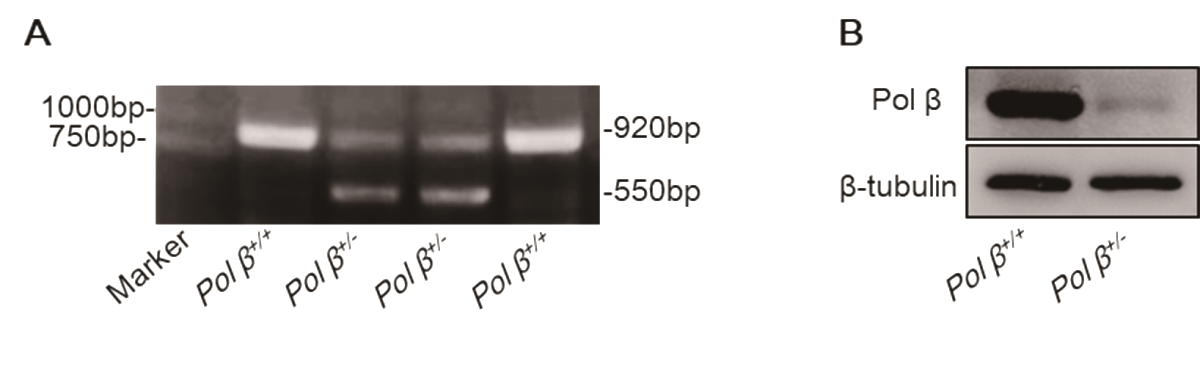


**Fig. S2. Establishment of *Pol β^+/-^* transgenic mice.** We created *Pol β^+/-^* mice by CRISPR/Cas9-mediated genome engineering, the *Pol β^+/-^* mice were identified by PCR and Western blotting. **(A)** Representative agarose gel electrophoresis of genomic DNA isolated from wild-type and *Pol β^+/-^* mice. **(B)** Representative blot of three independent experiments.

**
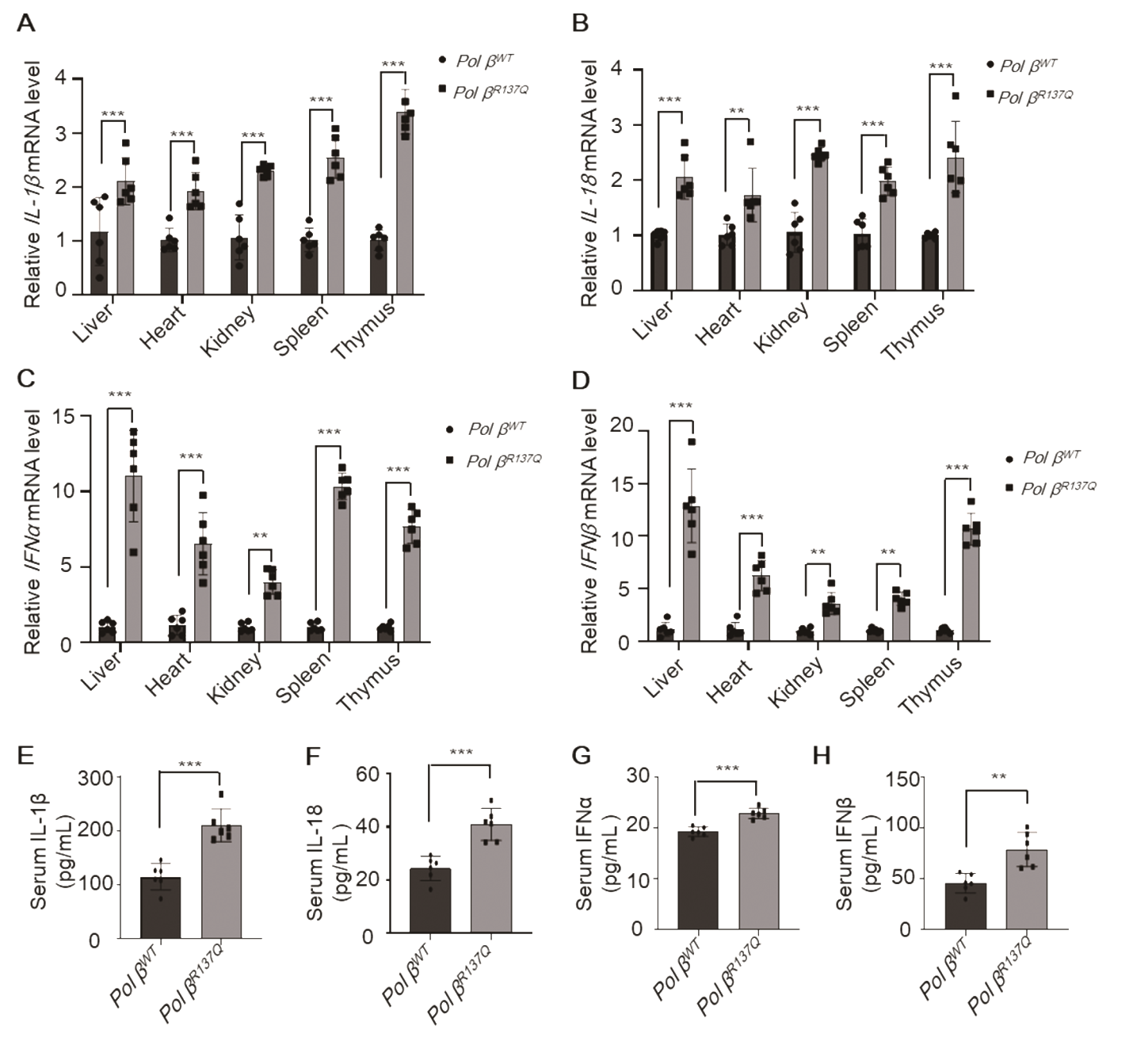
**

**Fig. S3. Pol β deficiency upregulates the levels of inflammatory cytokines in mice. (A-D)** The levels of IL-1β, IL-18, IFNα, IFNβ mRNA in the liver, heart, kidney spleen and thymus of mice were examined by qRT-PCR. **(E-H)** The protein levels of IL-1β, IL-18, IFNα, IFNβ in the serum of mice were analyzed by ELISA. ^**^*P*<0.01, ^***^*P*<0.001. Data present mean ± SD of at least 6 mice per group. A-D, two-way ANOVA, E-H, Student’s *t*-test.

**
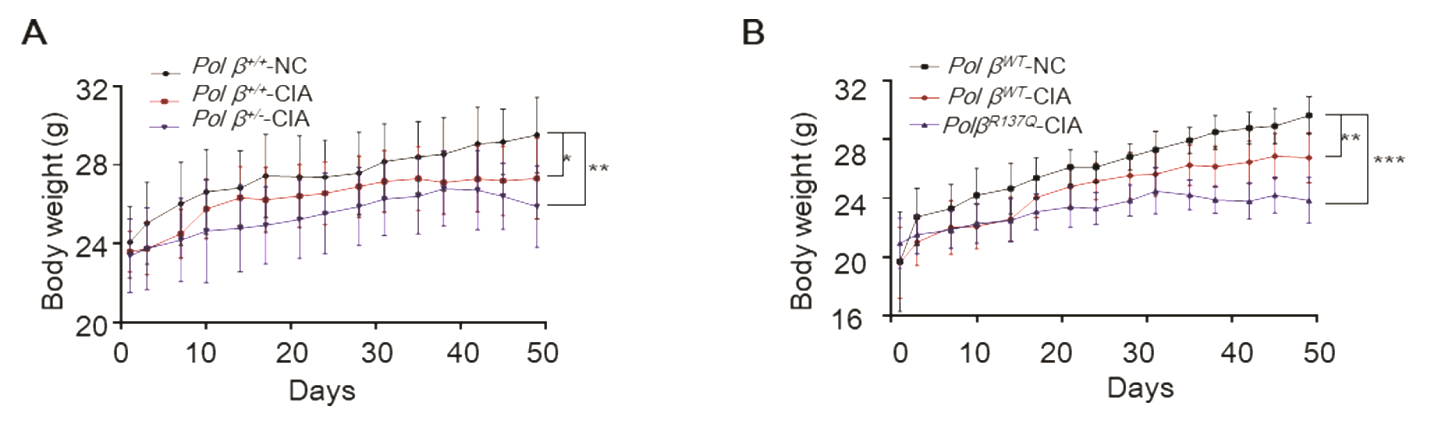
**

**Fig. S4. Pol β deficiency decreases the body weight of CIA mice. (A, B)** Body weight in C57BL/6J mice (Pol β^+/+^-NC, *Pol β^+/+^*-CIA, *Pol β^+/-^*-CIA) (A) and 129S1 mice (*Pol β^WT^*-NC, *Pol β^WT^*-CIA, *Pol β^R137Q^*-CIA) (B). ^*^*P*<0.05, ^**^*P*<0.01, ^***^*P*<0.001. Data present mean ± SD of at least 6 mice per group, two-way ANOVA.


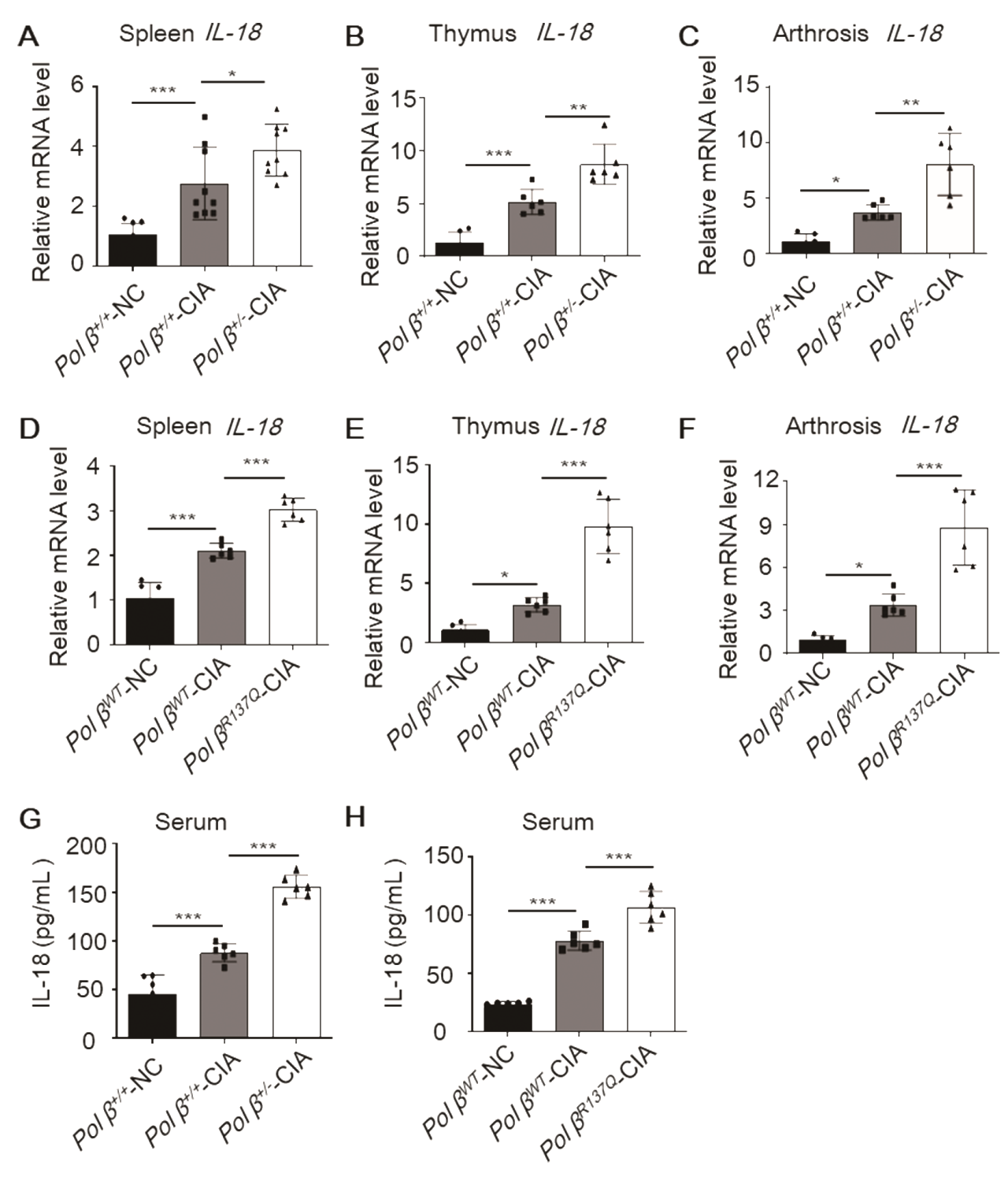


**Fig. S5. Pol β deficiency increases IL-18 in CIA mice. (A–F)** IL-18 mRNA levels in the spleen, thymus and arthrosis of CIA mice were detected by qRT-PCR. **(G, H)** The levels of IL-1β in the serum of CIA mice were analyzed by ELISA. Data are mean ± SD of at least 6 mice per group. ^*^*P*<0.05, ^**^*P*<0.01, ^***^*P*<0.001, one-way ANOVA. Data points indicate individual mice.


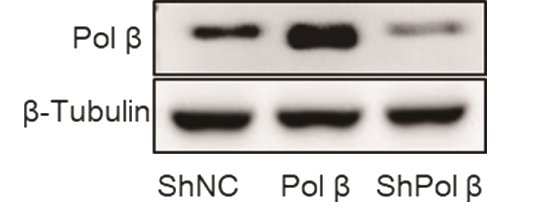


**Fig. S6. Establishment of stable Pol β knockdown or overpression RAW264.7 cell line.** RAW264.7 cells were transfected with ShNC, Pol β and ShPol β for 48 hours, and then the protein level of Pol β was checked by western blotting. β-tubulin was used as an internal control. Data are representative blot of three independent experiments.

**
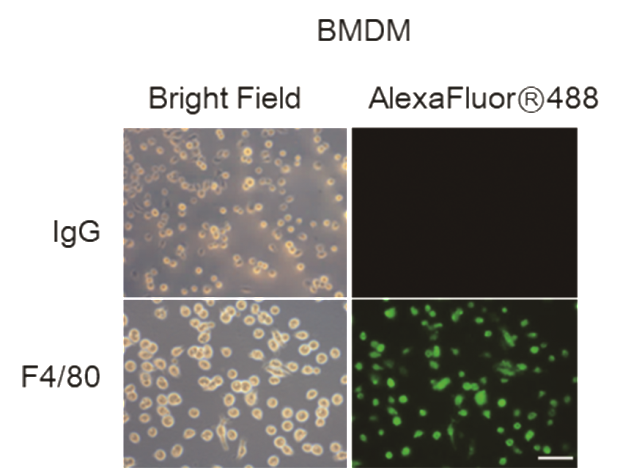
**

**Fig. S7. Primary mouse BMDM cells were identified by using anti-F4/80 antibody.** Representative images of primary mouse BMDM cells**.** Scale bar, 50 μm.

**
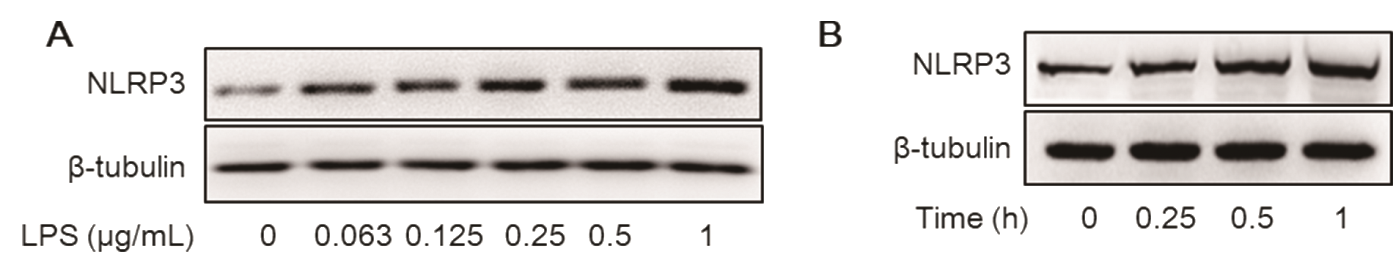
**

**Fig. S8. Macrophages pyroptosis induced by LPS plus ATP.** **(A)** RAW264.7 cells were treated with 0 - 1 μg/mL LPS for 5 hours, and NLRP3 expression levels were measured by Western blotting. **(B)** RAW264.7 cells were pretreated with 1 μg/mL LPS, and 5 mM ATP was then added and incubated for an additional 0 - 1 hour. The total incubation time was 5 hours, and the protein levels of NLRP3 were measured by Western blotting. A and B, representative blot of three independent experiments.


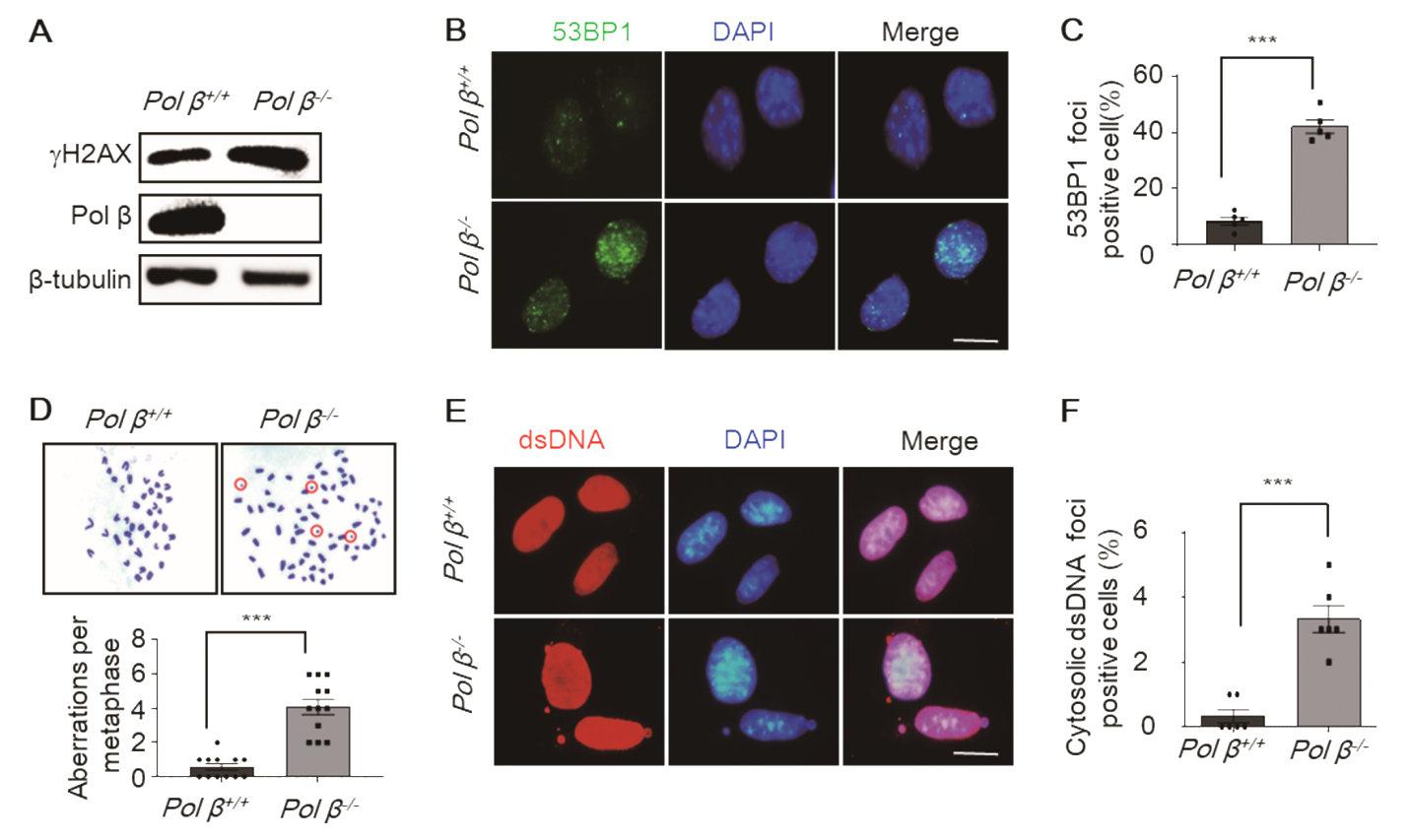


**Fig. S9. Damaged DNA accumulated in Pol β-deficient cells. (A)** Representative Western blot analyses of Pol β and γ-H2AX in Pol β-deficient MEF cells. **(B)** Representative immunofluorescence images of 53BP1 foci in cells. scale bar, 10 μm. **(C)** Statistical analysis of 53BP1 foci positive cell numbers in panel B. The data represent the mean ± SEM from more than 100 cells for each group; Student’s *t*-test, ^***^*P*<0.001. **(D)** The effect of Pol β deficiency on genomic instability was examined by karyotype analysis. Representative chromosome count images of metaphase cells were chosen and indicated chromosomal breakage. Lower panel: statistical analysis, data represent the mean ± SEM from more than 100 cells for each group, Student’s *t*-test, ^***^*P*<0.001. **(E)** Representative immunofluorescence images of dsDNA in *Pol β^+/+^* and *Pol β^-/-^* MEF cells. scale bar, 10 μm. **(F)** Statistical analysis of cytosolic dsDNA foci positive cells in panel E. The data represent the mean ± SEM from more than 100 cells for each group; Student’s *t*-test, ^***^*P*<0.001.


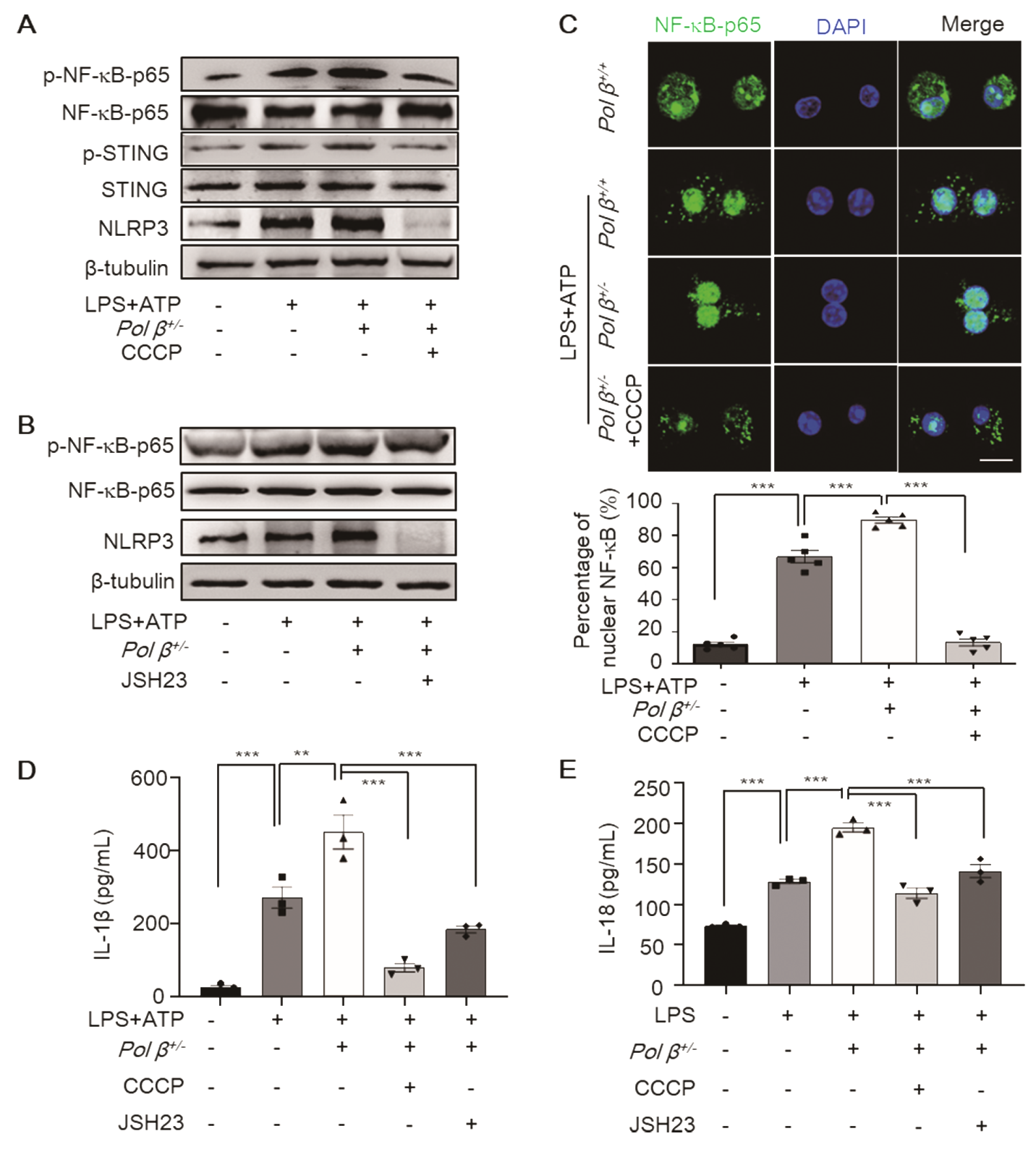


**Fig. S10. Pol β deficiency exacerbates pyroptosis in BMDMs via the cGAS/STING/NF-κB pathway.** Pol β-deficient BMDMs were pretreated with 10 μM CCCP or 10 μM JSH-23 for 2 hours, treated with 1 μg/mL LPS for 4 h, and then treated with 5 mM ATP for the final hour. **(A, B)** The levels of STING, p-STING, NF-κB-p65, p-NF-κB-p65, and NLRP3 were determined by Western blot, β-tubulin was used as an internal control. Data are representative blot of three independent experiments. **(C)** Representative images of NF-κB-p65 staining. Scale bar, 10 μm. Lower panel: statistical analysis, the data represent the mean ± SEM from more than 100 cells for each group; ^***^*P*<0.001, one-way ANOVA. **(D, E)** The levels of IL-1β and IL-18 secreted by macrophages were analyzed by ELISA. Data present the mean ± SEM of three independent experiments, ^**^*P*<0.01, ^***^*P*<0.001, one-way ANOVA.
