# Supplementary material for: A novel mechanism for macrophage pyroptosis in rheumatoid arthritis induced by Pol β deficiency
Source: Cell Death Dis. 2022 Jul 6;13(7):583. doi: 10.1038/s41419-022-05047-6 (PMC9259649; doi:10.1038/s41419-022-05047-6)
Supplement: Supplementary file 2 — original western blots [file 41419_2022_5047_MOESM2_ESM.pdf]

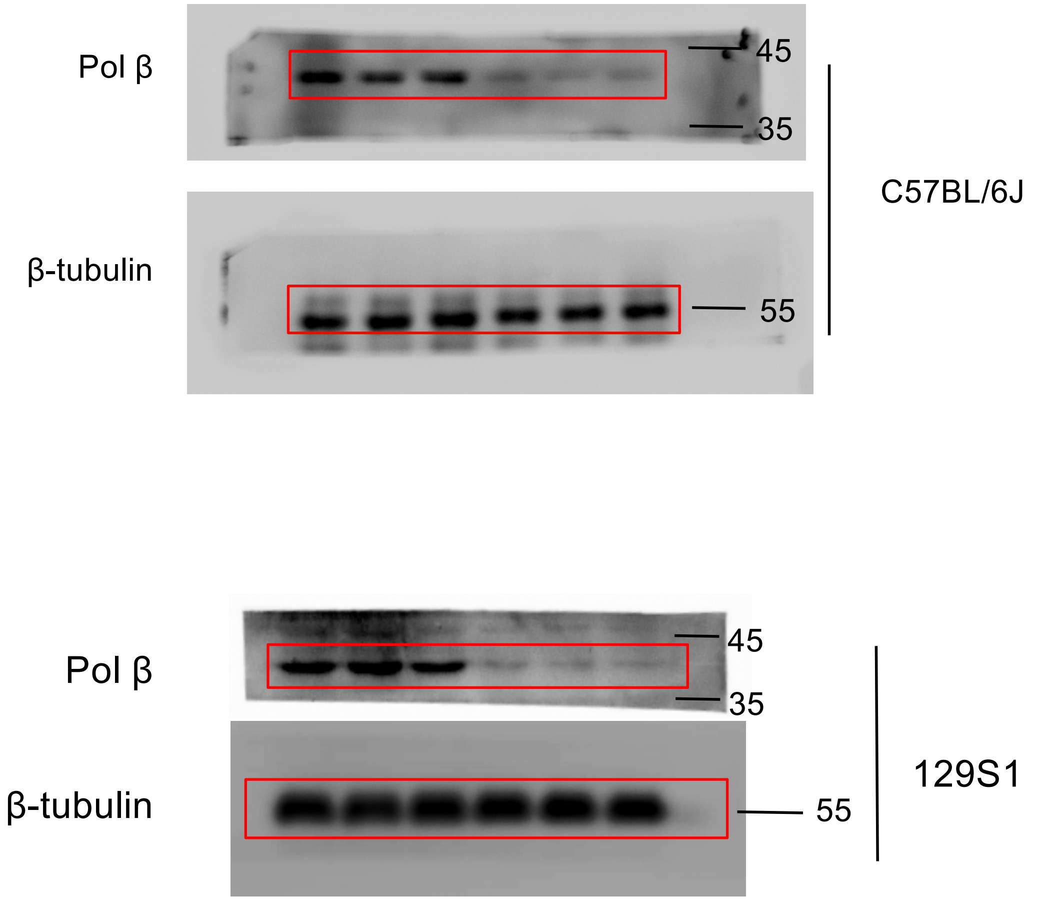

Figure 1E

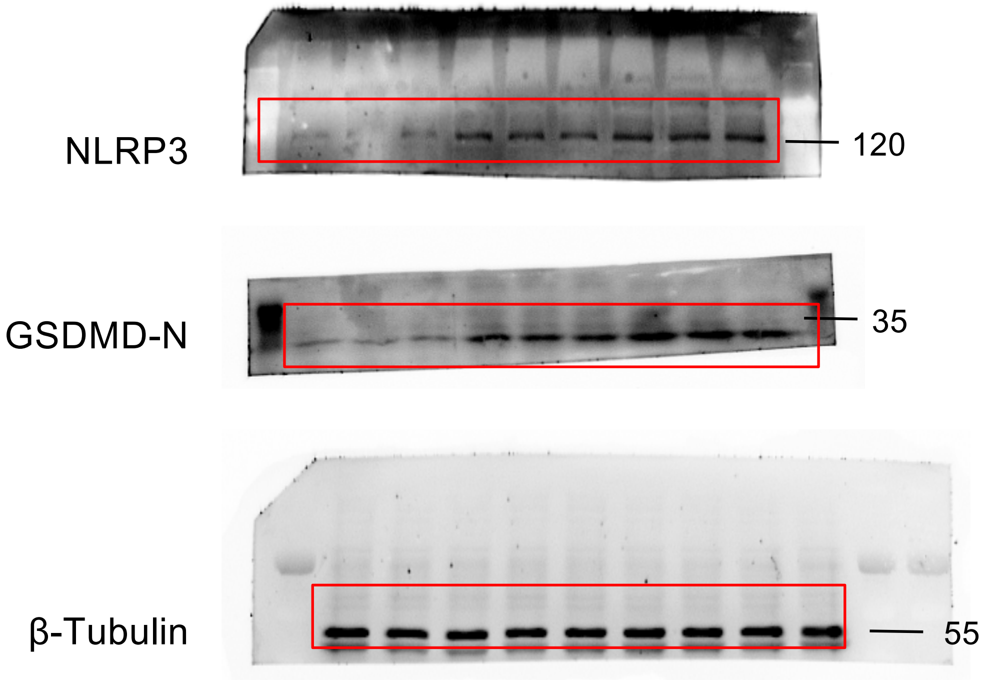

Figure 2K

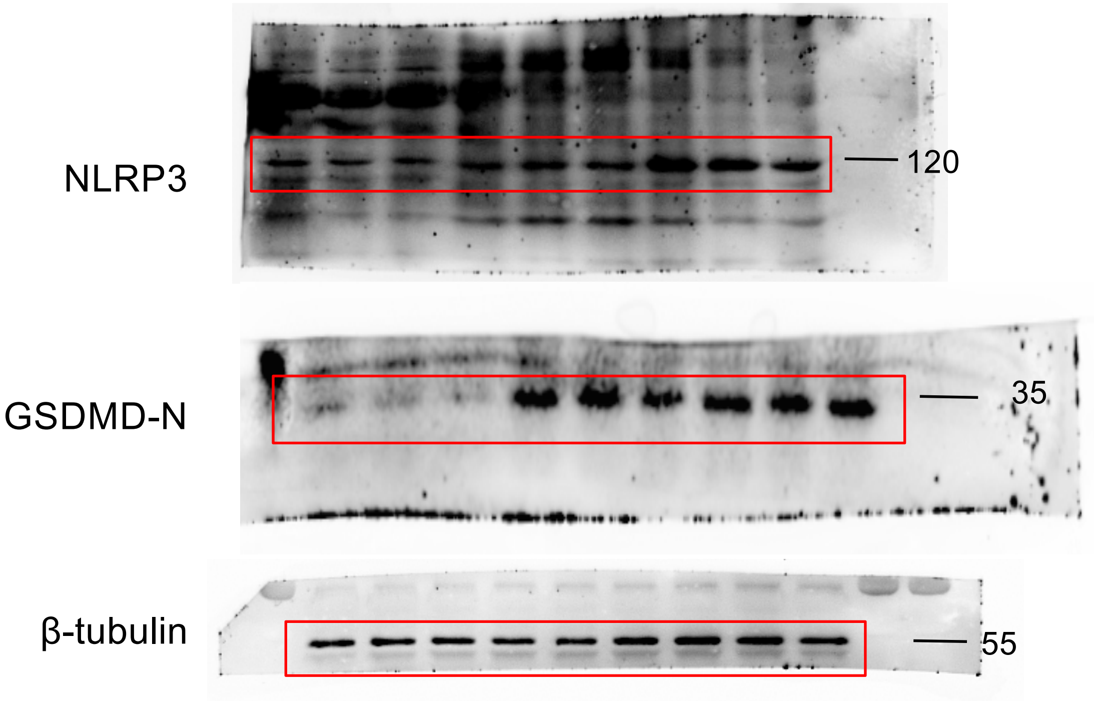

Figure 2L

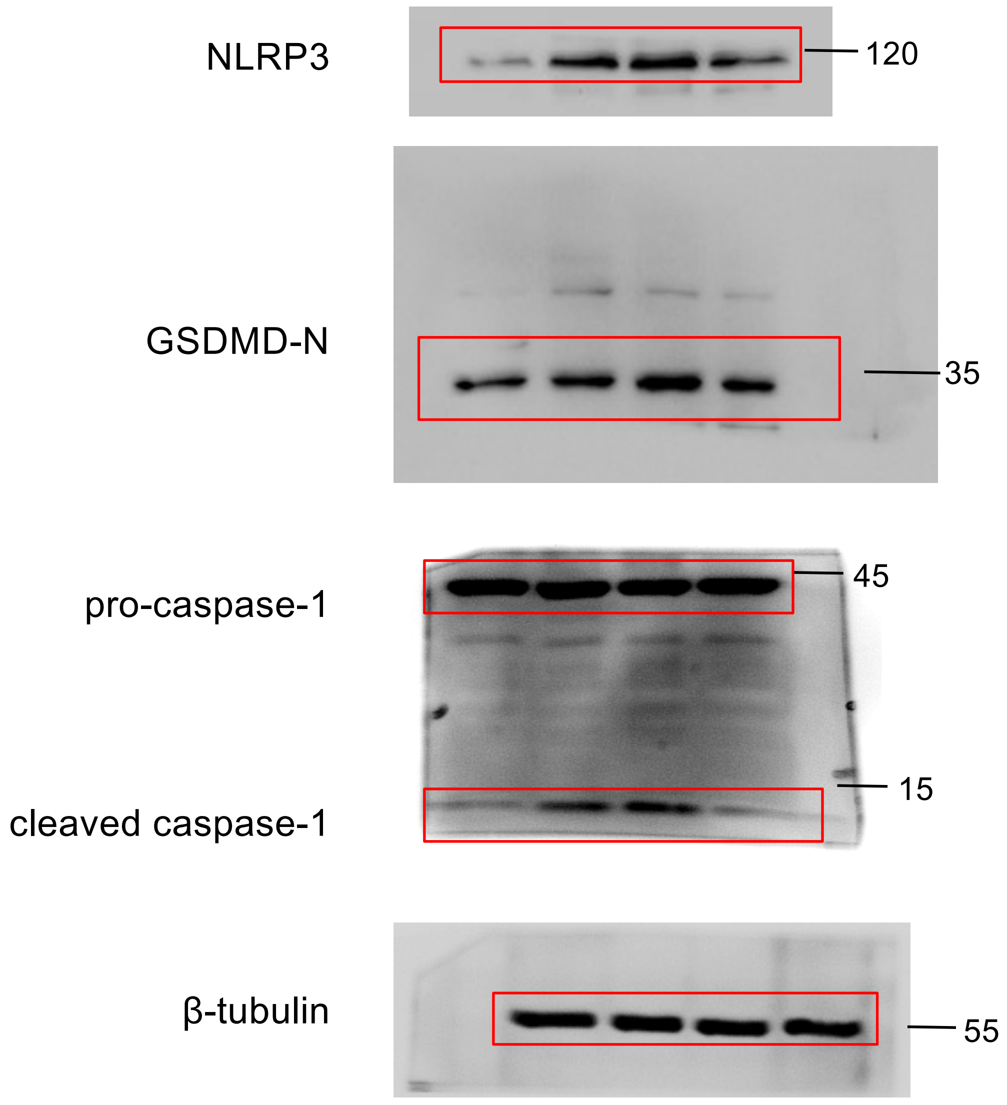
NLRP3
120
GSDMD-N
35
pro-caspase-1
45
15
cleaved caspase-1
β-tubulin
55

Figure 3A

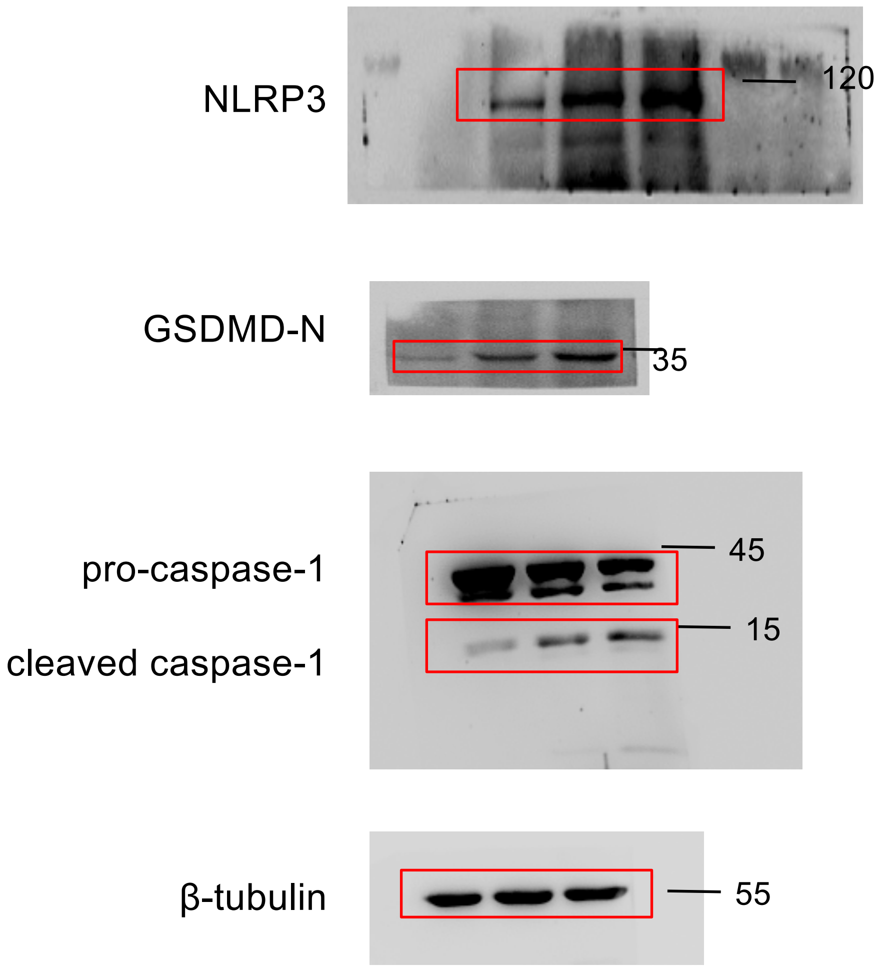
NLRP3
120
GSDMD-N
35
pro-caspase-1
45
cleaved caspase-1
15
β-tubulin
55

Figure 3B

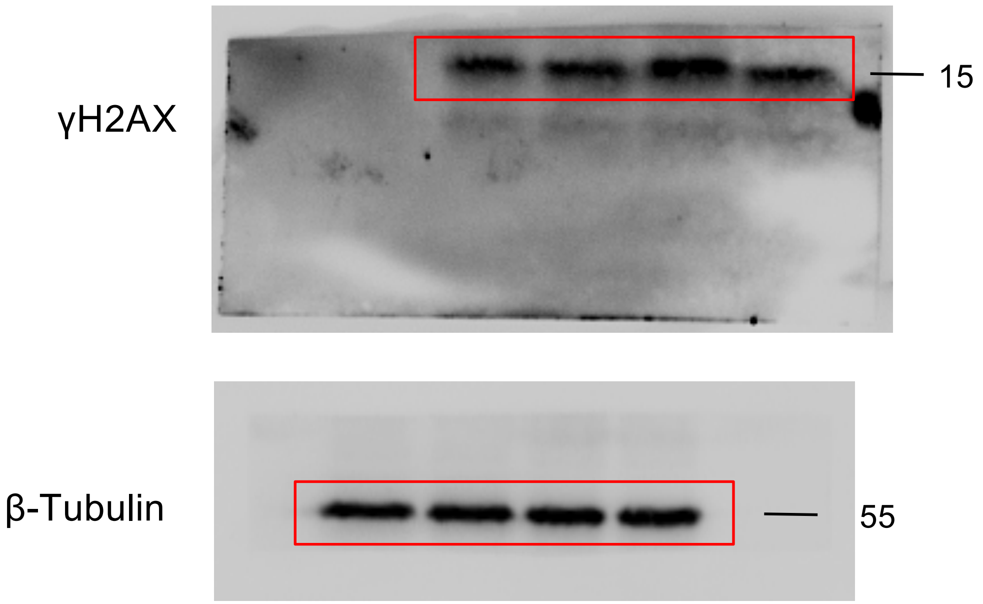

Figure 4C

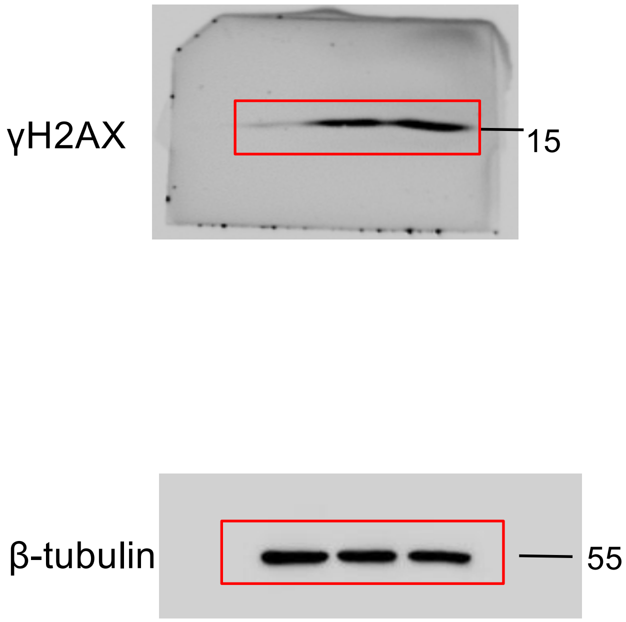

Figure 4F

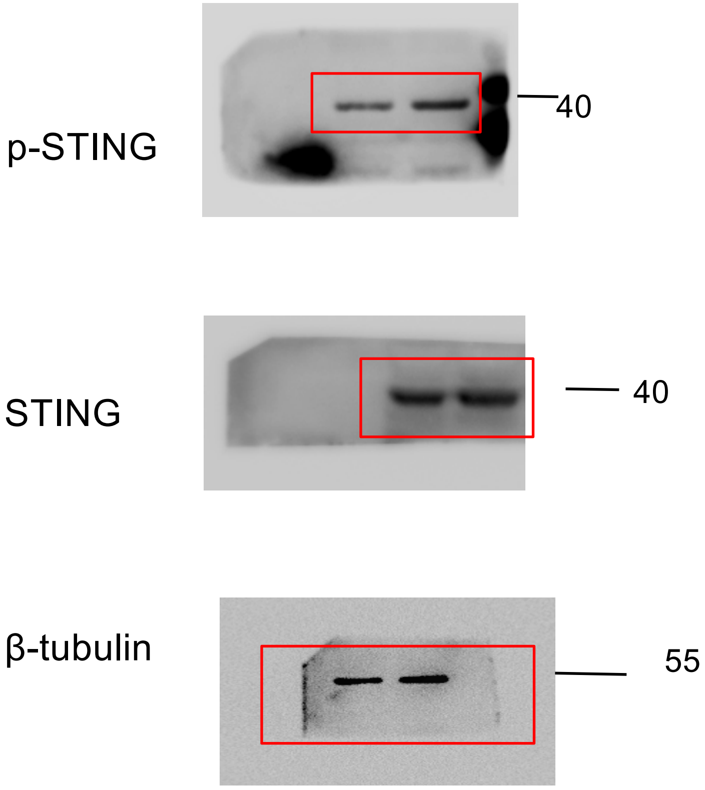

Figure 6B

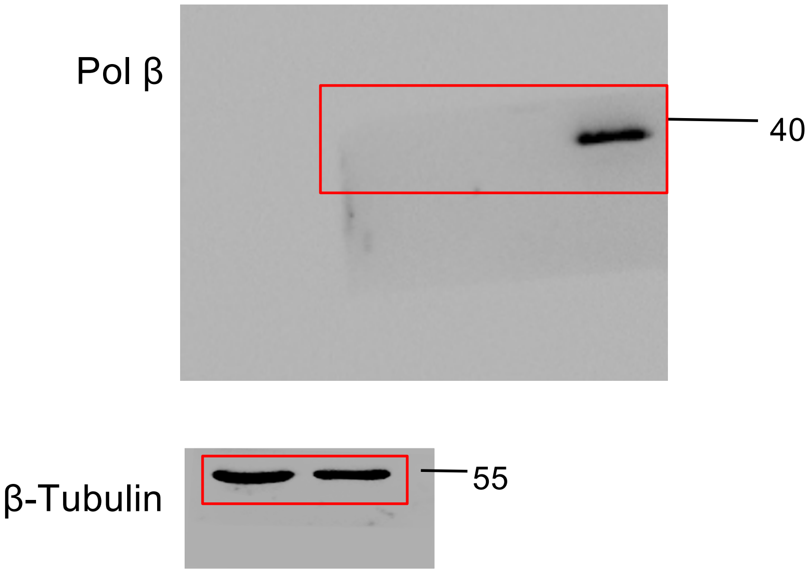

Figure 6D

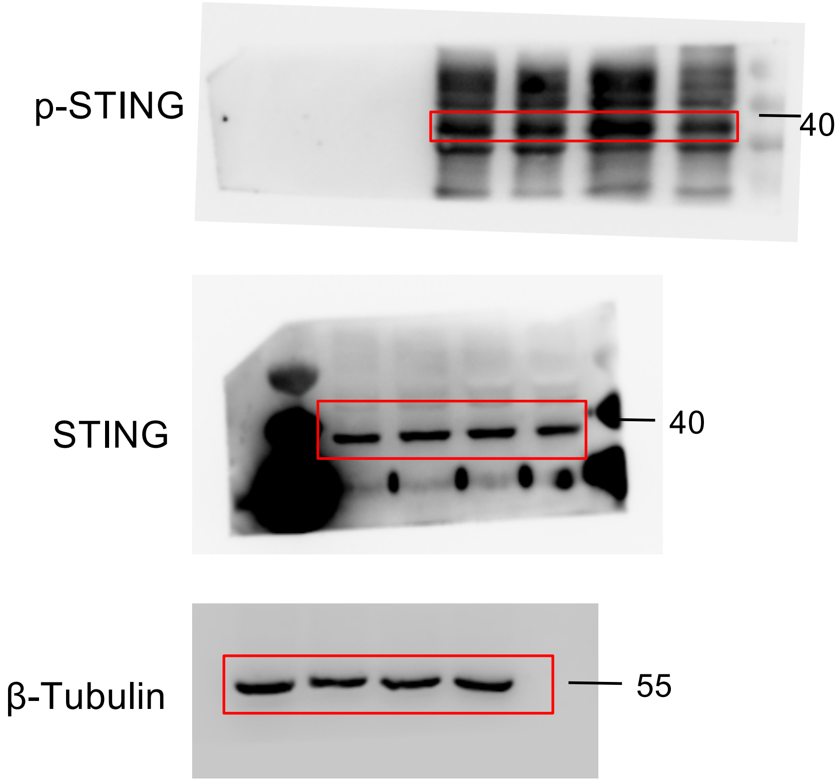

Figure 6F

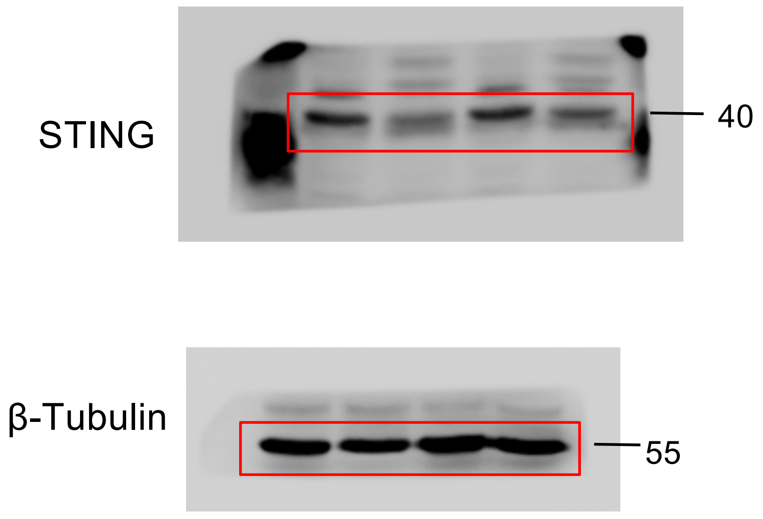

Figure 6H

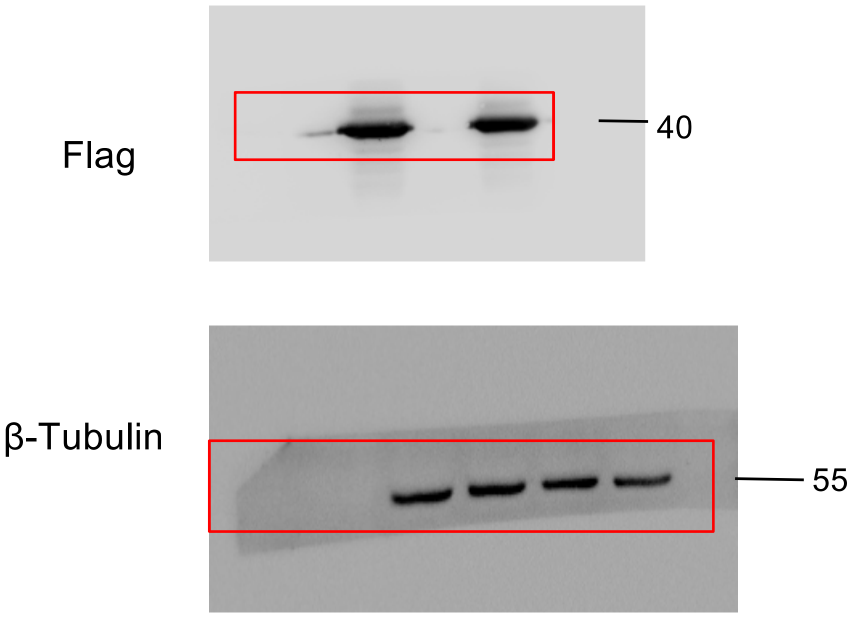

Figure 6J

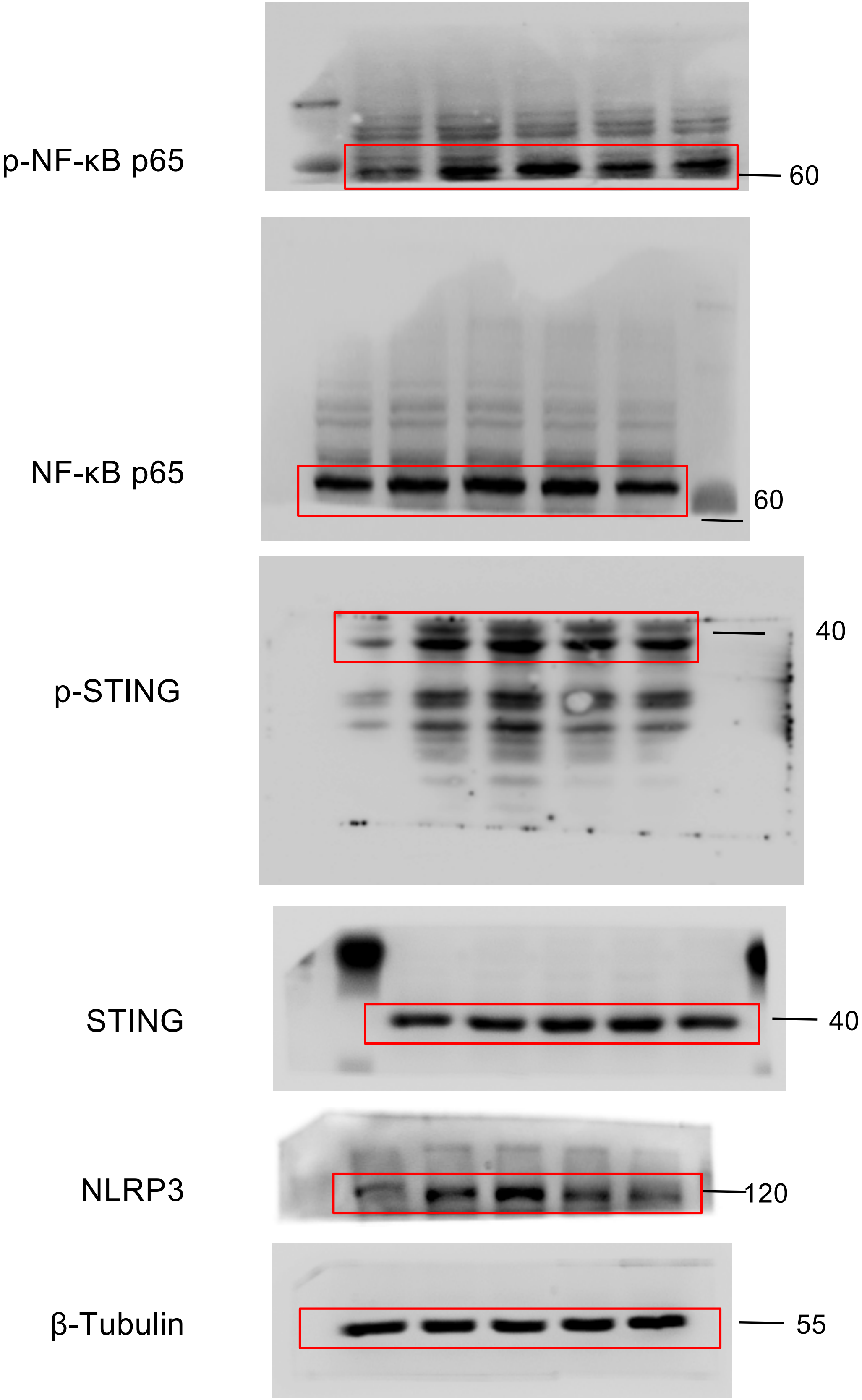
p-NF-κB p65
60
NF-κB p65
60
p-STING
40
STING
40
NLRP3
120
β-Tubulin
55

Figure 7A

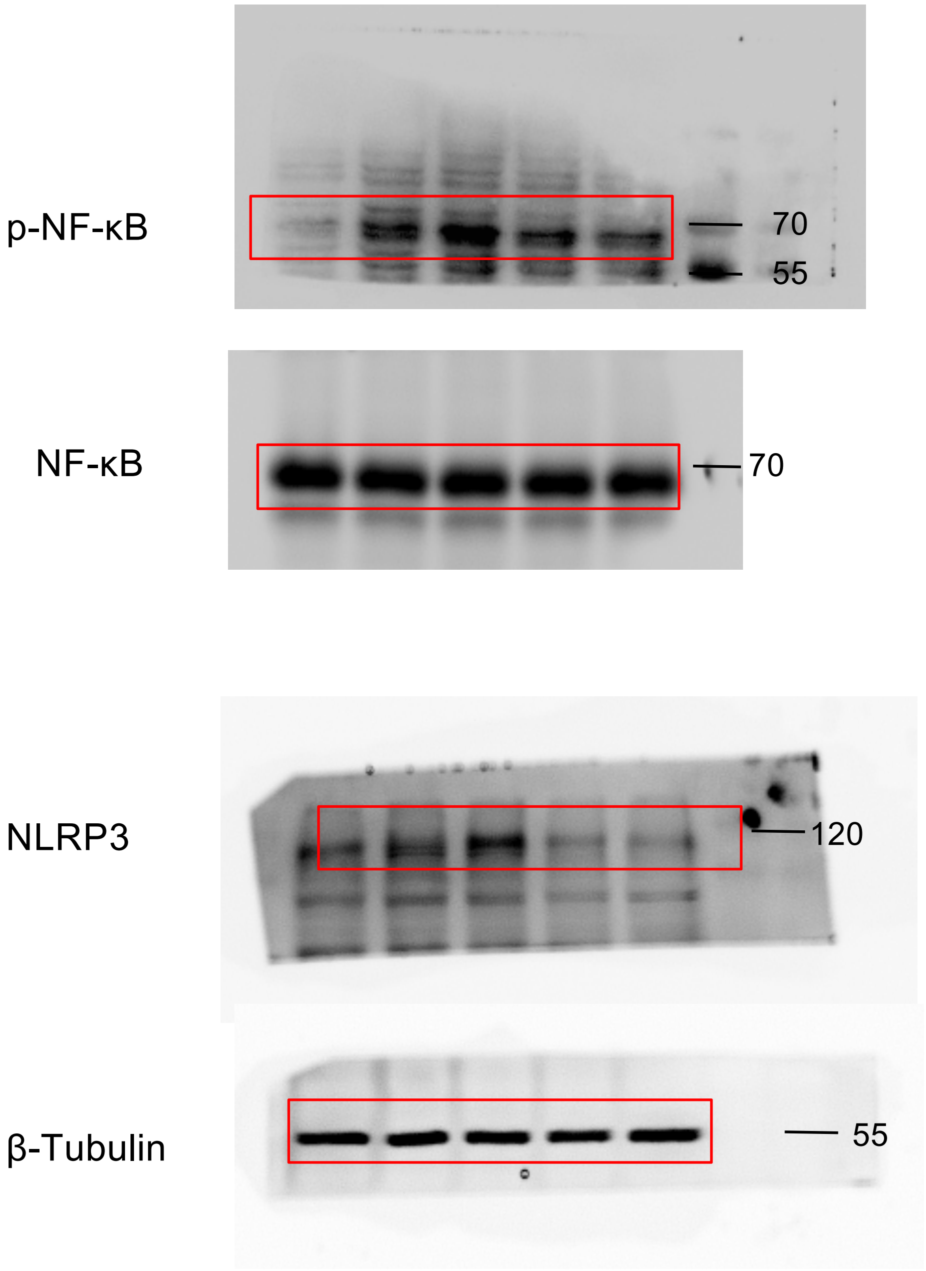

Figure 7B

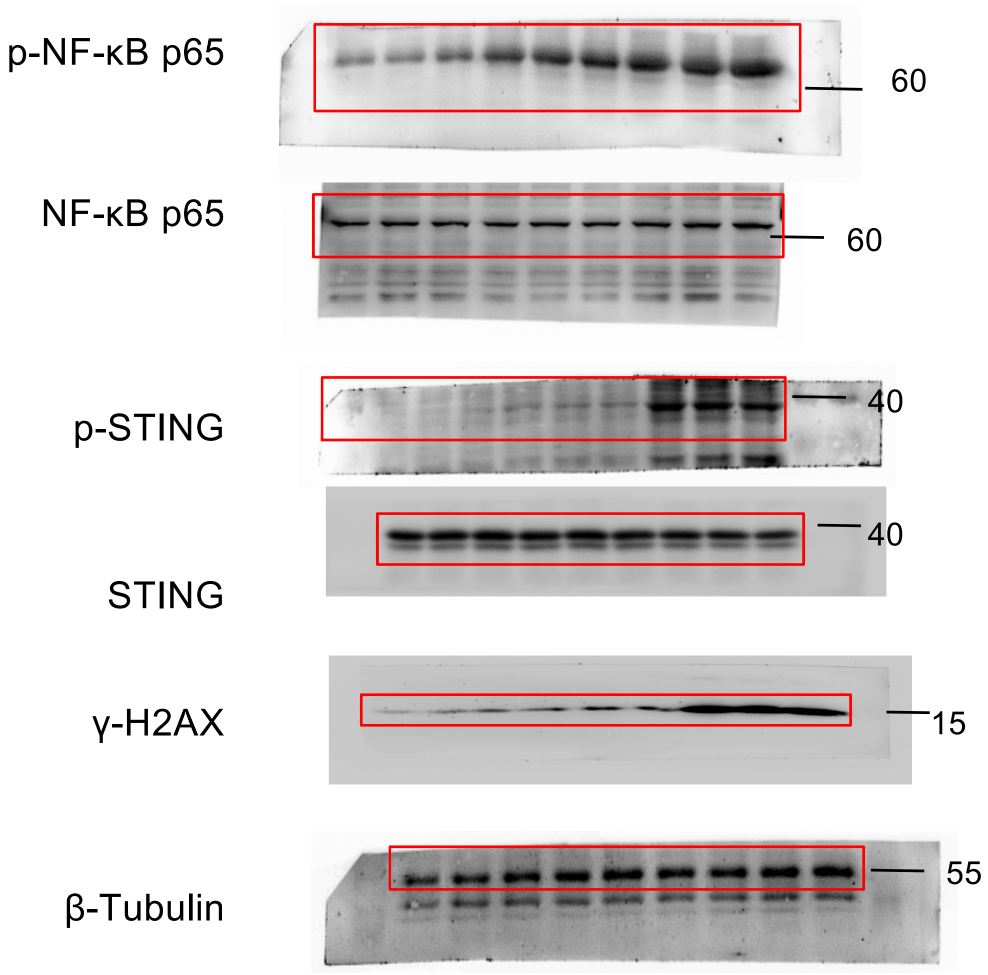

p-NF-κB p65
60
NF-κB p65
60
p-STING
40
40
STING
γ-H2AX
15
β-Tubulin
55

Figure 7F

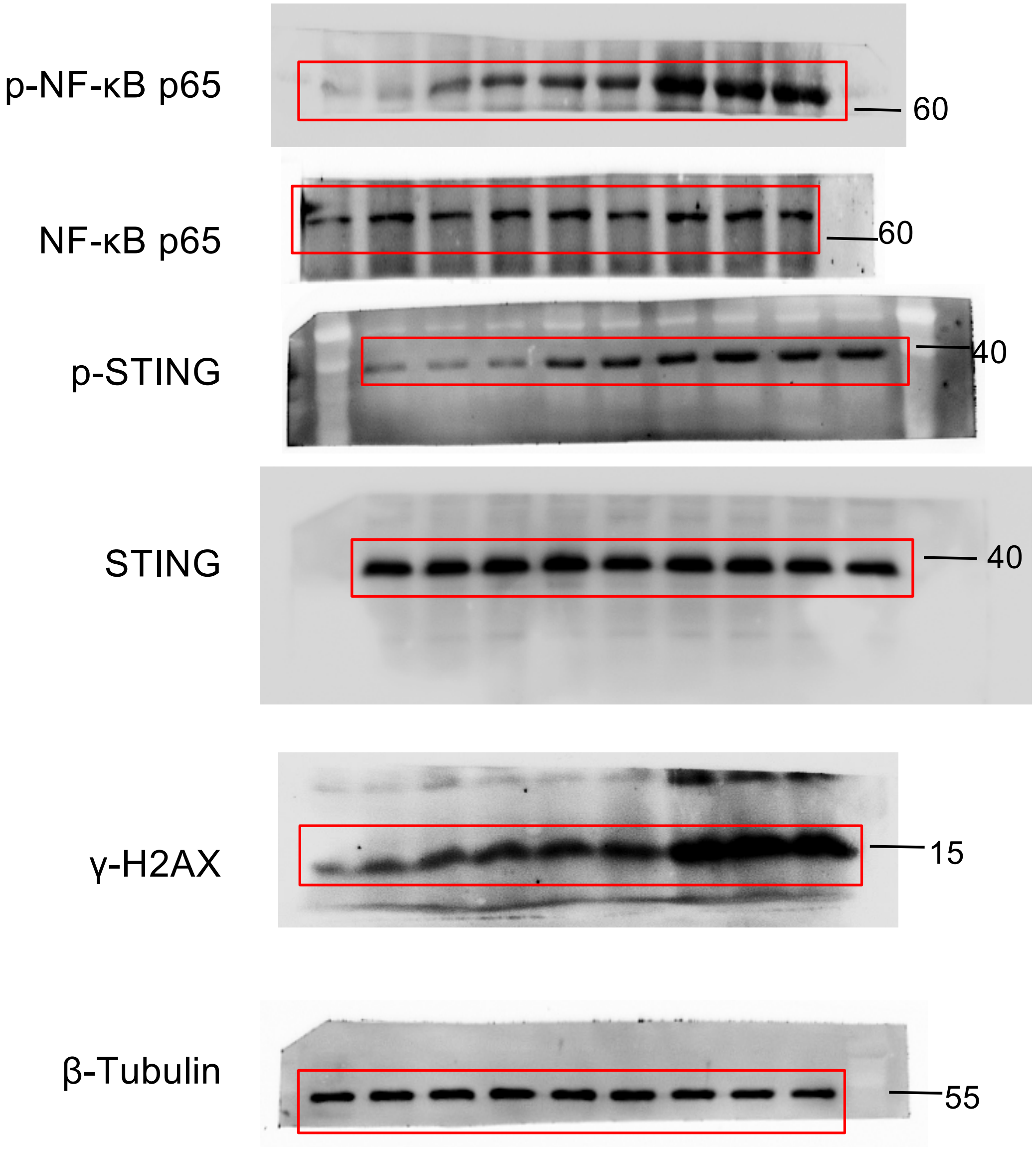
p-NF-κB p65
60
NF-κB p65
60
p-STING
40
STING
40
γ-H2AX
15
β-Tubulin
55

Figure 7G

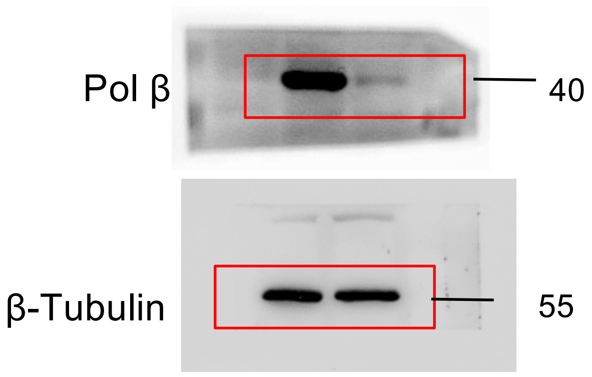

Figure S2

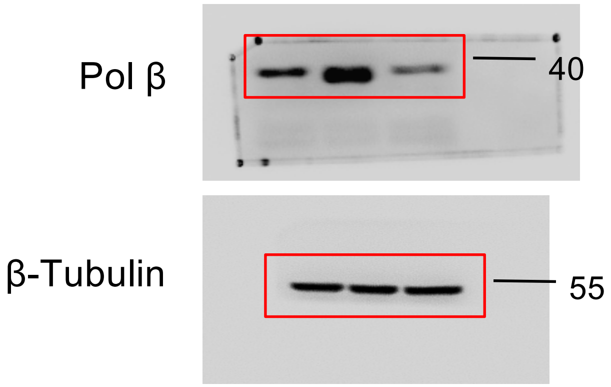

Figure S6

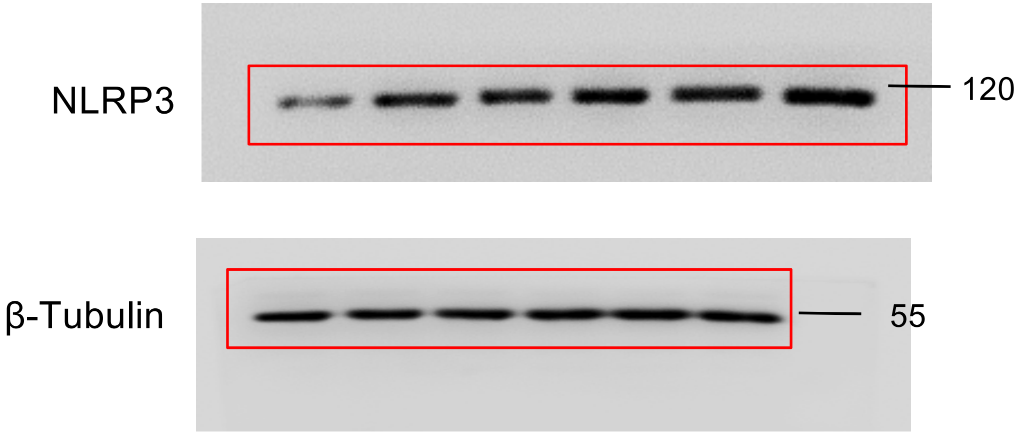

Figure S8A

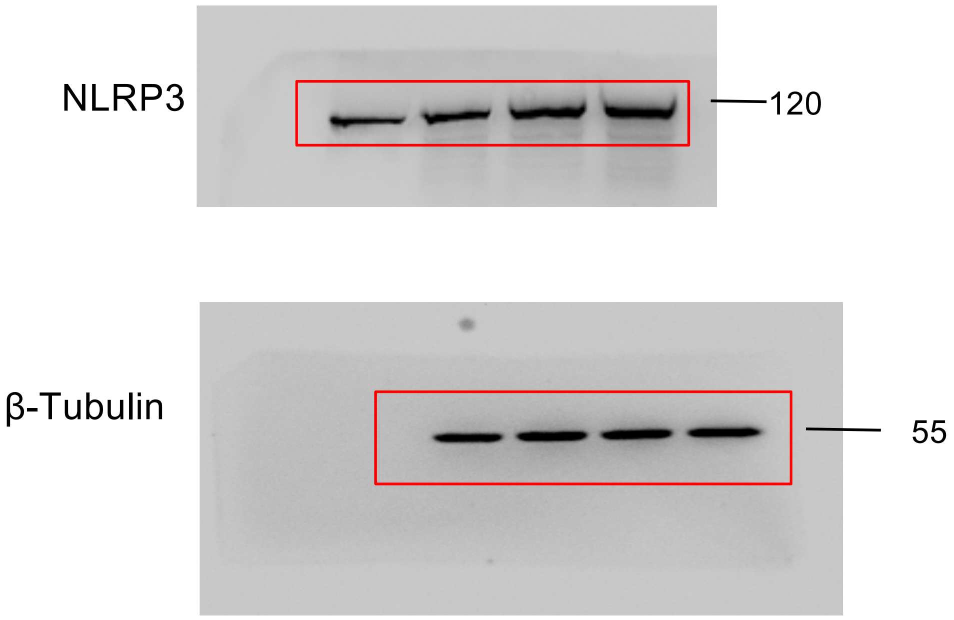

Figure S8B

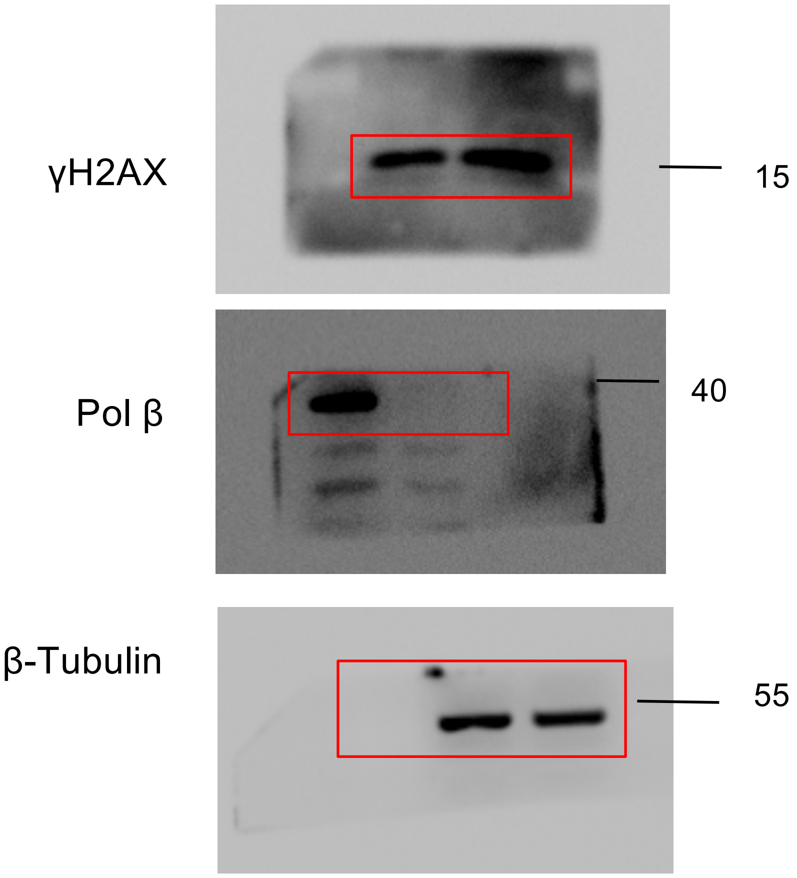

Figure S9A

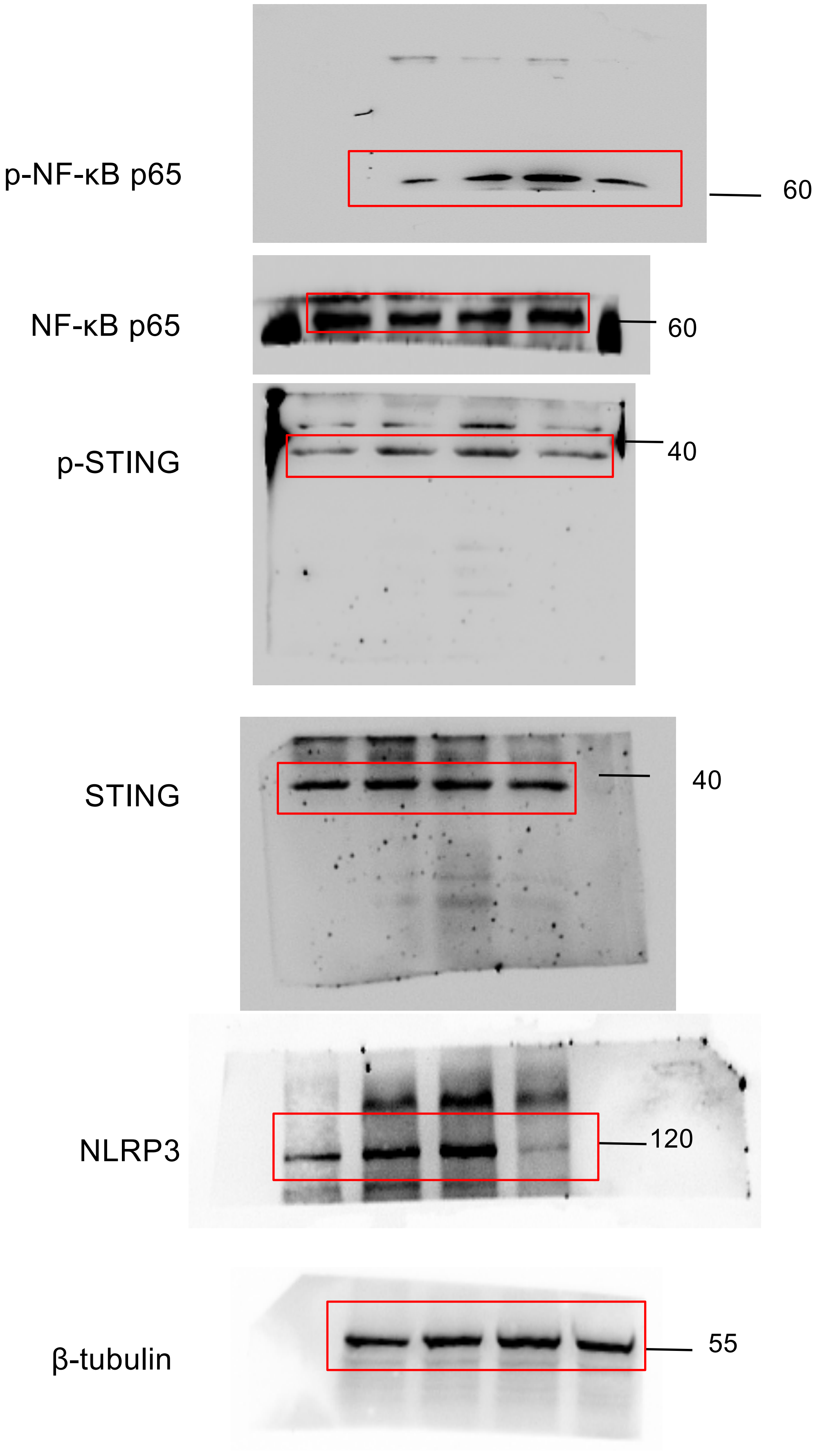
p-NF-κB p65
60
NF-κB p65
60
p-STING
40
STING
40
NLRP3
120
β-tubulin
55

Figure S10A

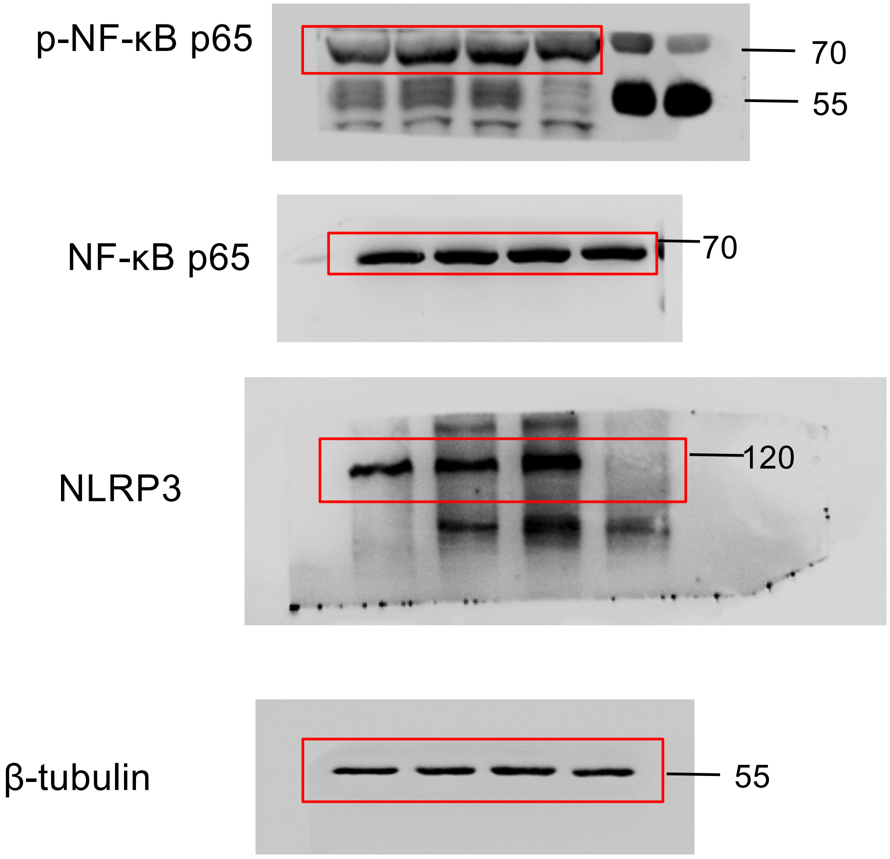

Figure S10B
