# Supplementary material for: A novel mechanism for macrophage pyroptosis in rheumatoid arthritis induced by Pol β deficiency
Source: Cell Death Dis. 2022 Jul 6;13(7):583. doi: 10.1038/s41419-022-05047-6 (PMC9259649; doi:10.1038/s41419-022-05047-6)
Supplement: Supplementary file 3 — aj-checklist [file 41419_2022_5047_MOESM3_ESM.pdf]

Corresponding Author Name: Zhigang Guo
Manuscript Number: CDDIS-22-0252R

SPRINGER NATURE

Reporting Summary

Springer Nature wishes to improve the reproducibility of the work that we publish. This checklist is used to ensure good reporting standards and to improve the reproducibility. Please respond completely to all questions relevant to your manuscript. For more information, please read the journal's Guide to Authors.

☑ **Check here to confirm that the following information is available in the Material & Methods section:**

- **The exact sample size (*n*)** for each experimental group/condition, given as a number, not a range
- **A description of the sample collection** allowing the reader to understand whether the samples represent technical or biological replicates (including how many animals, litters, culture, etc.)
- **A statement of how many times the experiment shown was replicated in the laboratory**
- **Definitions of statistical methods and measures**: For small sample sizes (n<5) descriptive statistics are not appropriate, instead plot individual data points
    - Very common tests, such as *t*-test, simple χ^2 tests, Wilcoxon and Mann-Whitney tests, can be unambiguously identified by name only, but more complex techniques should be described in the methods section
    - Are tests one-sided or two-sided?
    - Are there adjustments for multiple comparisons?
    - **Statistical test results**, e.g., ***P* values**
    - Definition of **'center values'** as **median or mean**;
    - Definition of **error bars as s.d. or s.e.m. or c.i.**

Please ensure that the answers to the following questions are reported in the manuscript itself. We encourage you to include a specific subsection in the methods section for statistics, reagents and animal models. Below, provide the page number or section and paragraph number.

Statistics and general methods	Reported in section/paragraph or page #
1. How was the sample size chosen to ensure adequate power to detect a pre-specified effect size? (Give section/paragraph or page #)	The animal samples collected were presented in each figure legend and method section. Cell lines were described in the method section. All the experiments were repeated three
For animal studies, include a statement about sample size estimate even if no statistical methods were used.	No. We have presented animal sample sizes in each figure legend and method section.
2. Describe inclusion/exclusion criteria if samples or animals were excluded from the analysis. Were the criteria pre-established? (Give section/paragraph or page #)	No data were excluded from the analyses. No criteria were pre-established. All the animals experiments are described in the method section and figure legends.
3. If a method of randomization was used to determine how samples/animals were allocated to experimental groups and processed, describe it. (Give section/paragraph or page #)	Samples/animals were assigned randomly to the various experimental groups. All these experiments are described in the method section.
For animal studies, include a statement about randomization even if no randomization was used.	All the animals were assigned randomly and processed.

4. If the investigator was blinded to the group allocation during the experiment and/or when assessing the outcome, state the extent of blinding. (Give section/paragraph or page #)	Arthritic scores and body weight were determined by two independent, blinded observers twice per week.
For animal studies, include a statement about blinding even if no blinding was done.	Yes, we have included the statement in the manuscript.
5. For every figure, are statistical tests justified as appropriate?	For every figure, the statistical tests are justified as appropriate.
Do the data meet the assumptions of the tests (e.g., normal distribution)?	Yes, our data met the assumptions.
Is there an estimate of variation within each group of data?	The variation within each group was calculated as standard deviation.
Is the variance similar between the groups that are being statistically compared? (Give section/paragraph or page #)	Yes, the variance is similar between the groups which are being statistically compared.

Reagents

	**Reported in section/paragraph or page #**
6. Report the source of antibodies (vendor and catalog number)	The source of all antibodies were reported in the Materials and Methods section.
7. Identify the source of cell lines and report if they were recently authenticated (e.g., by STR profiling) and tested for mycoplasma contamination	RAW264.7 were purchased from China Infrastructure of Cell Line Resources and MEFs were a gift from Prof. Binghui Shen (City of Hope), and authenticated by these providers, respectively. Cells were tested to be mycoplasma

Animal Models

	**Reported in section/paragraph or page #**
8. Report species, strain, sex and age of animals	The species, strain, sex and age of animals were reported in
9. For experiments involving live vertebrates, include a statement of compliance with ethical regulations and identify the committee(s) approving the experiments.	We reported a statement of compliance with ethical regulations and identify the committee(s) approving the experiments in the method section.

10. We recommend consulting the ARRIVE guidelines (*PLoS Biol.* **8**(6), e1000412,2010) to ensure that other relevant aspects of animal studies are adequately reported.

Human subjects

Reported in section/paragraph or page #

11. Identify the committee(s) approving the study protocol.	The ethics committee of The Affiliated Drum Tower Hospital of Nanjing University Medical School have approved the
12. Include a statement confirming that informed consent was obtained from all subjects.	The statement has been included in the Ethics Statement Section.
13. For publication of patient photos, include a statement confirming that consent to publish was obtained.	This work did not involve such data.
14. Report the clinical trial registration number (at ClinicalTrials.gov or equivalent).	This work did not involve clinical trial.

15. For phase II and III randomized controlled trials, please refer to the CONSORT statement and submit the CONSORT checklist with your submission.

16. For tumor marker prognostic studies, we recommend that you follow the REMARK reporting guidelines.

Data deposition

Reported in section/paragraph or page #

17. Provide accession codes for deposited data. Data deposition in a public repository is mandatory for:<br>a. Protein, DNA and RNA sequences<br>b. Macromolecular structures<br>c. Crystallographic data for small molecules<br>d. Microarray data	This work did not contain any deposited data.

Deposition is strongly recommended for many other datasets for which structured public repositories exist; more details on our data policy are available in the Guide to Authors. We encourage the provision of other source data in supplementary information or in unstructured repositories such as Figshare and Dryad. We encourage publication of Data Descriptors (see Scientific Data) to maximize data reuse.

18. If computer code was used to generate results that are central to the paper's conclusions, include a statement in the Methods section under "**Code availability**" to indicate whether and how the code can be accessed. Include version information as necessary and any restrictions on availability.	This work did not contain any computer code.
