# Supplementary material for: A novel mechanism for macrophage pyroptosis in rheumatoid arthritis induced by Pol β deficiency
Source: Cell Death Dis. 2022 Jul 6;13(7):583. doi: 10.1038/s41419-022-05047-6 (PMC9259649; doi:10.1038/s41419-022-05047-6)

Lili Gu


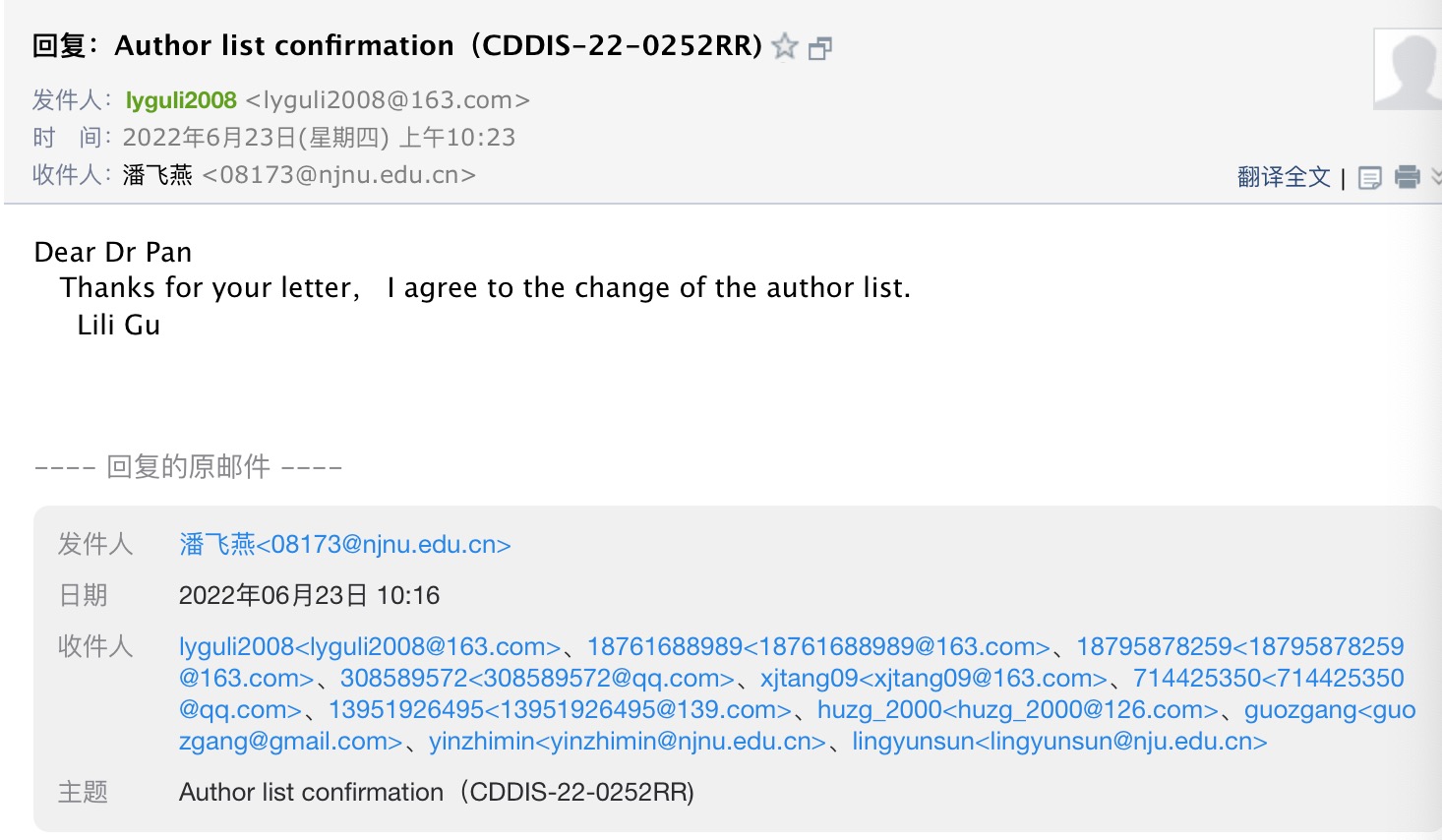


Yuling Sun


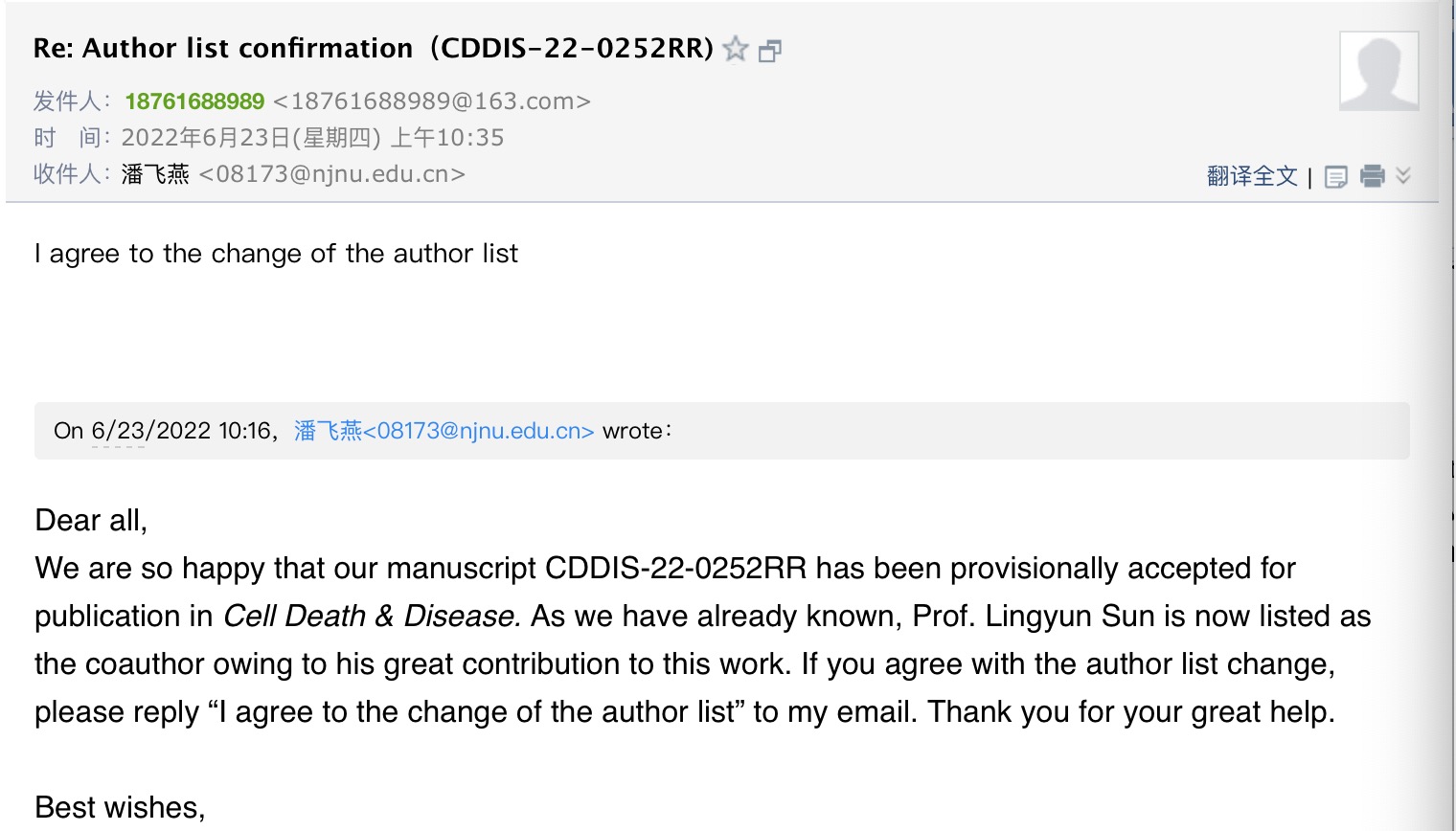


Ting Wu


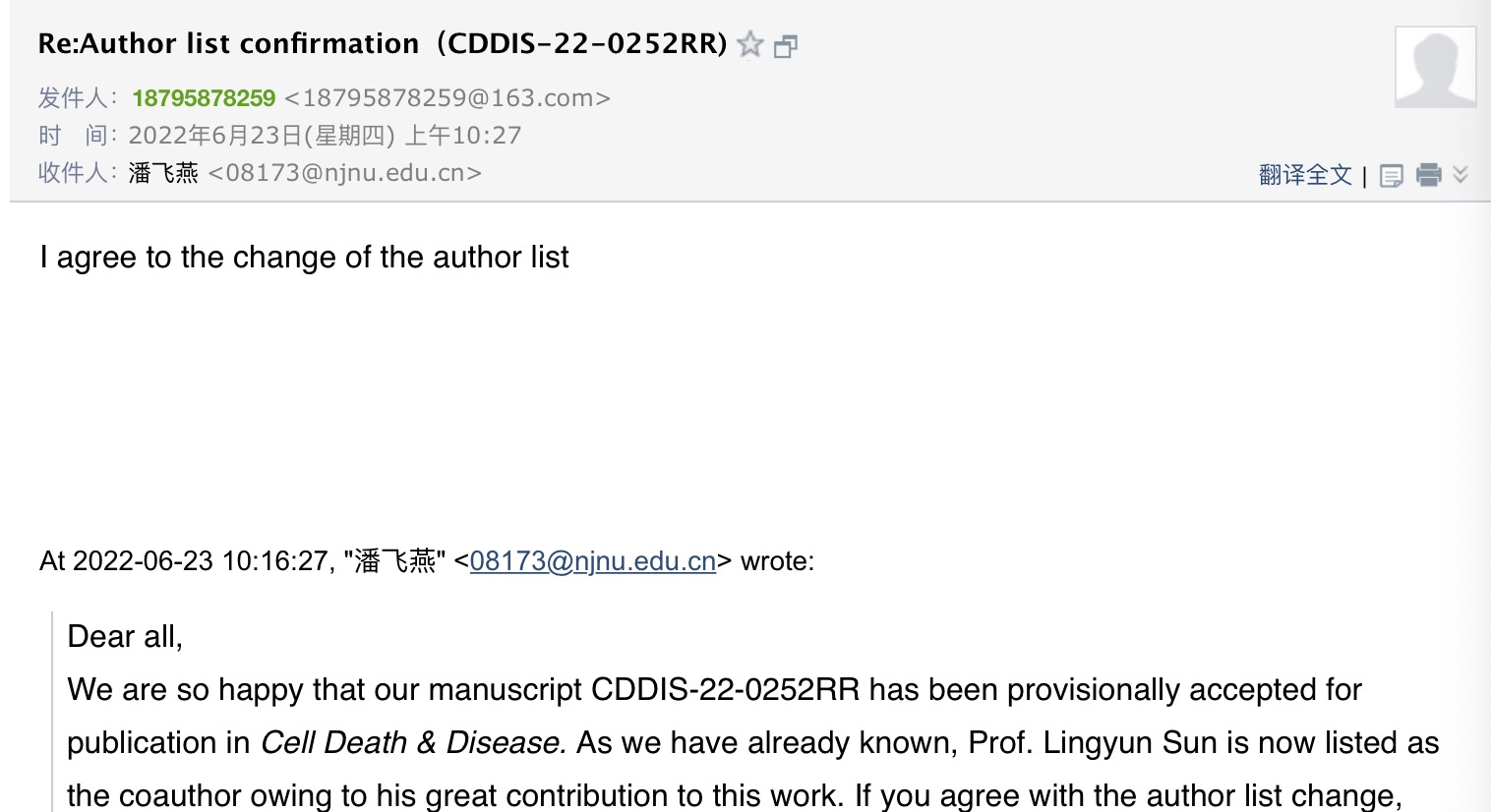


Ge Chen


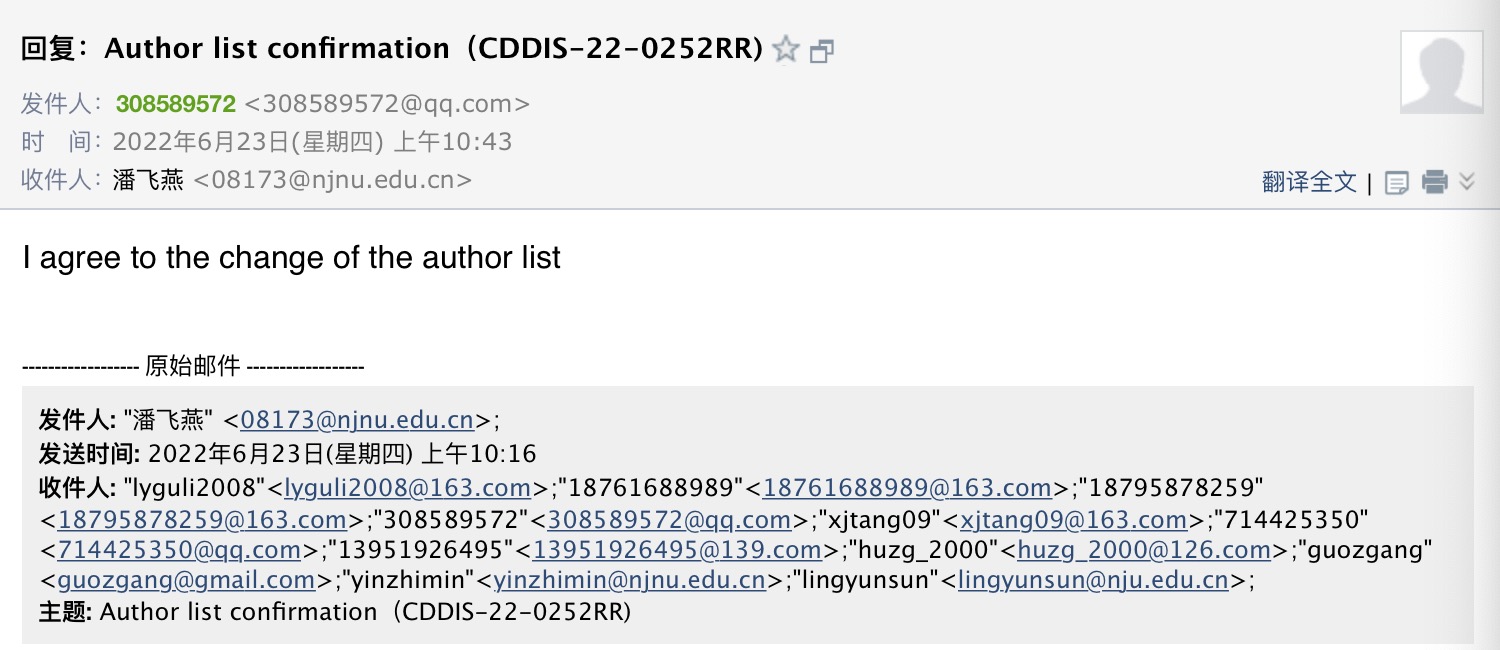


Xiaojun Tang


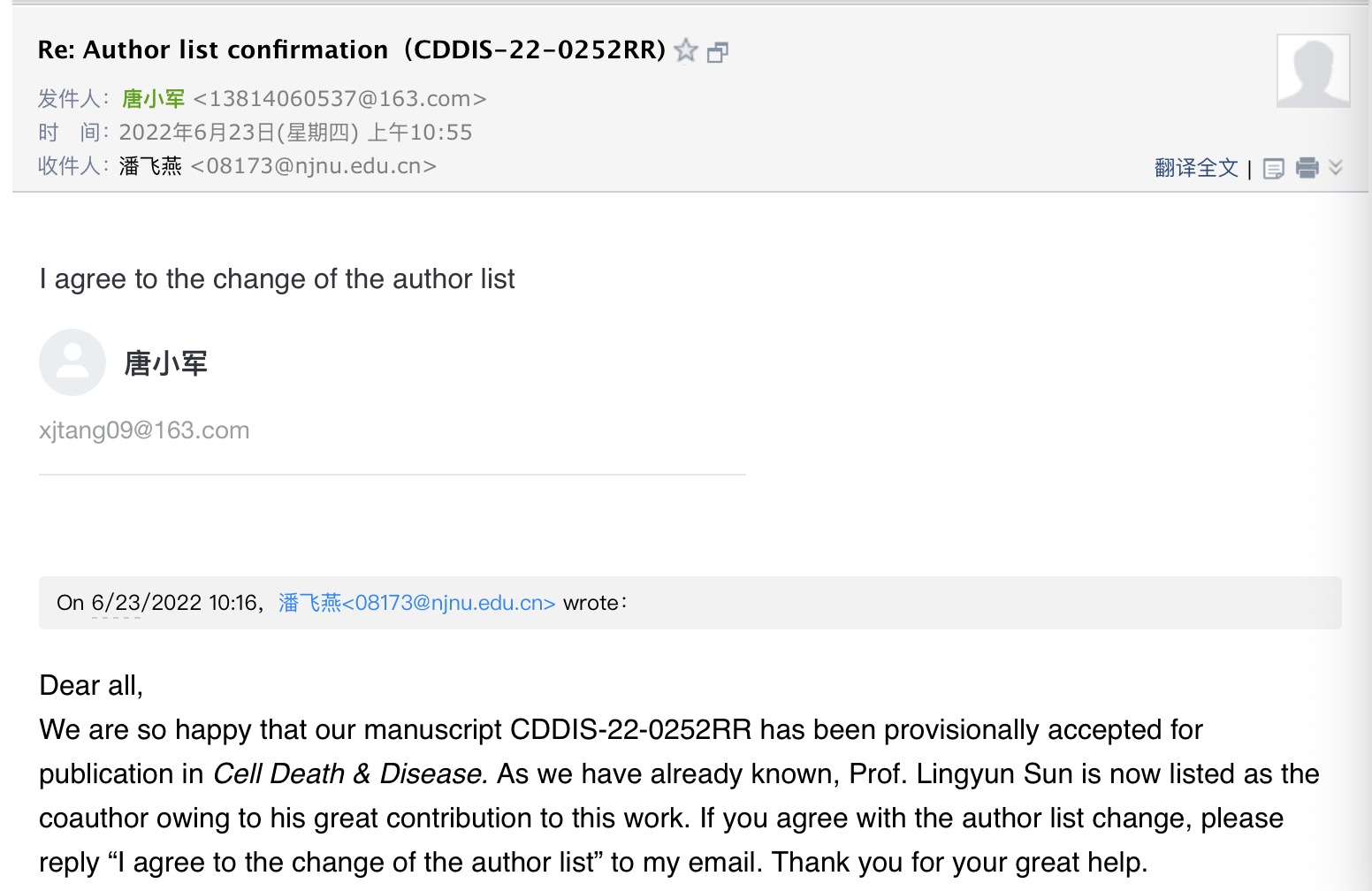


Lianfeng Zhao


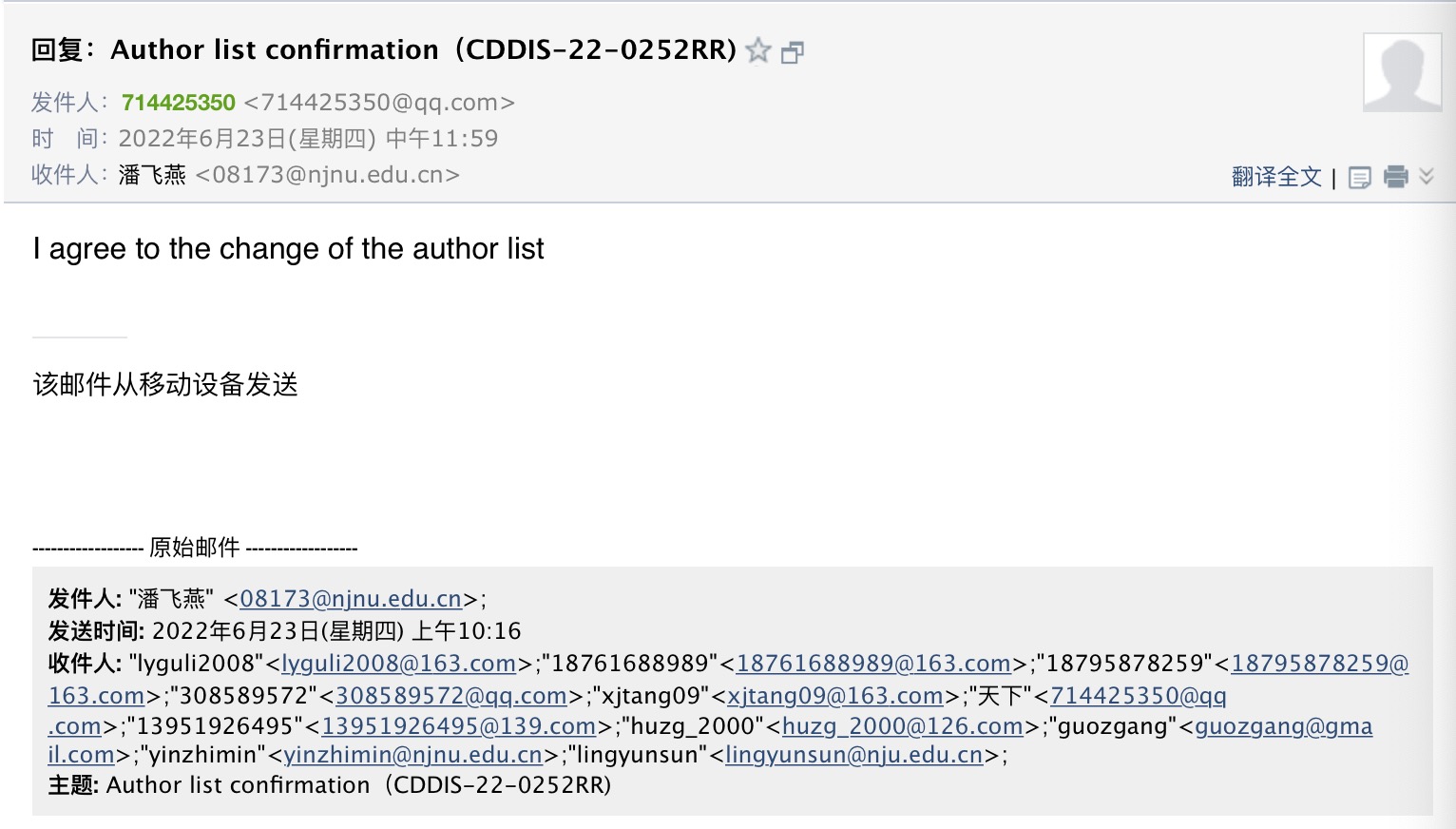


Lingfeng He


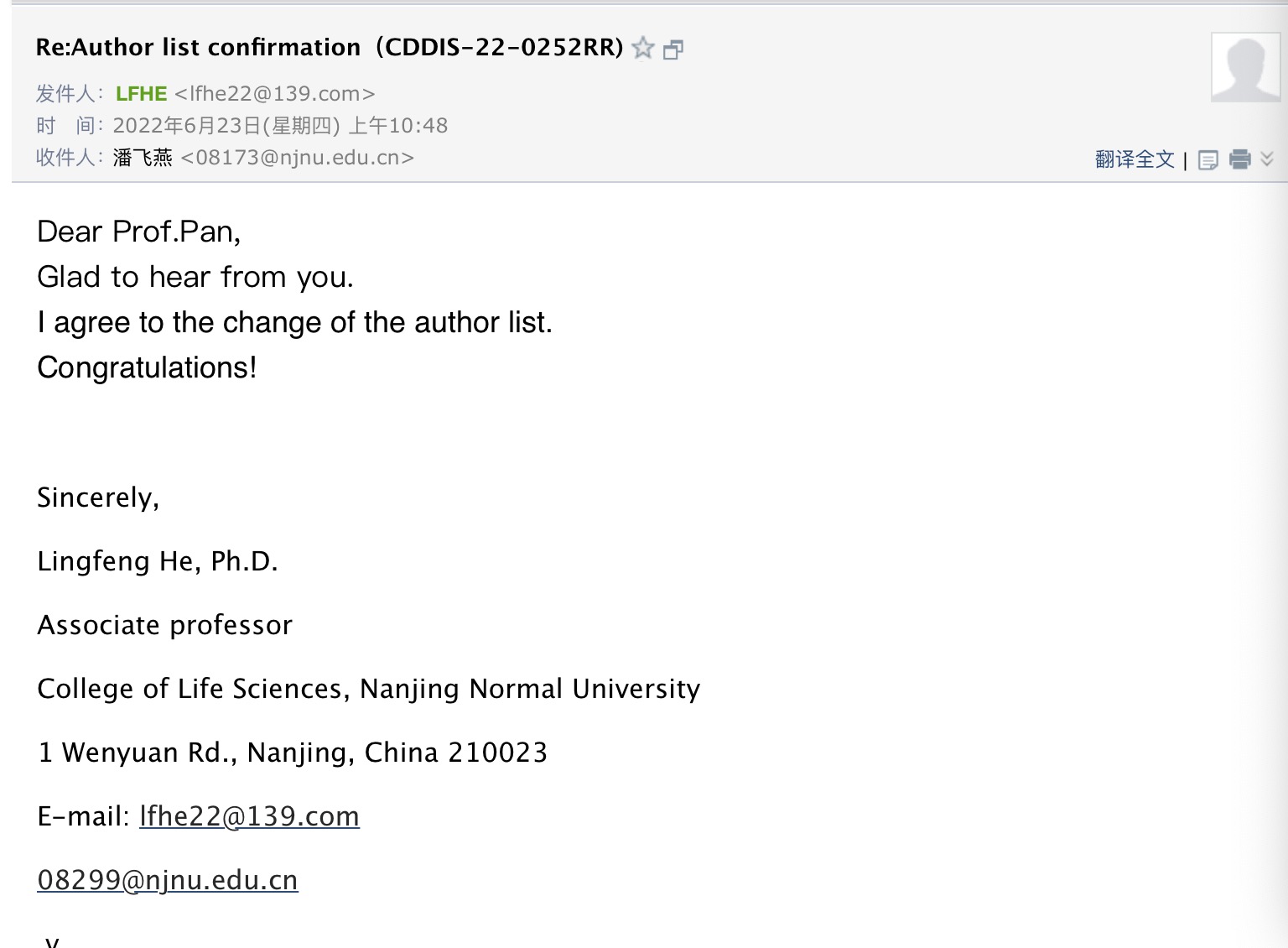


Zhigang Hu


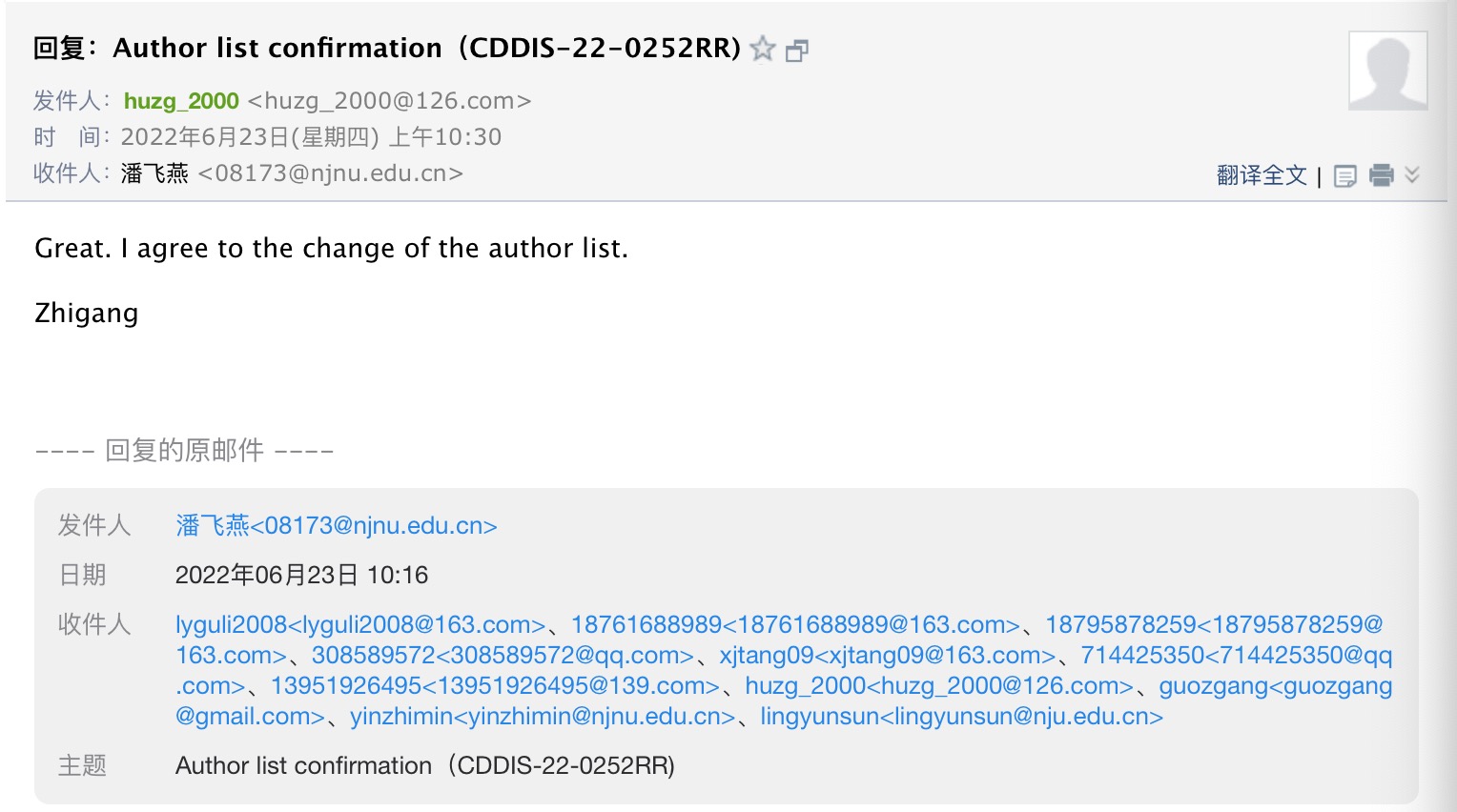


Lingyun Sun


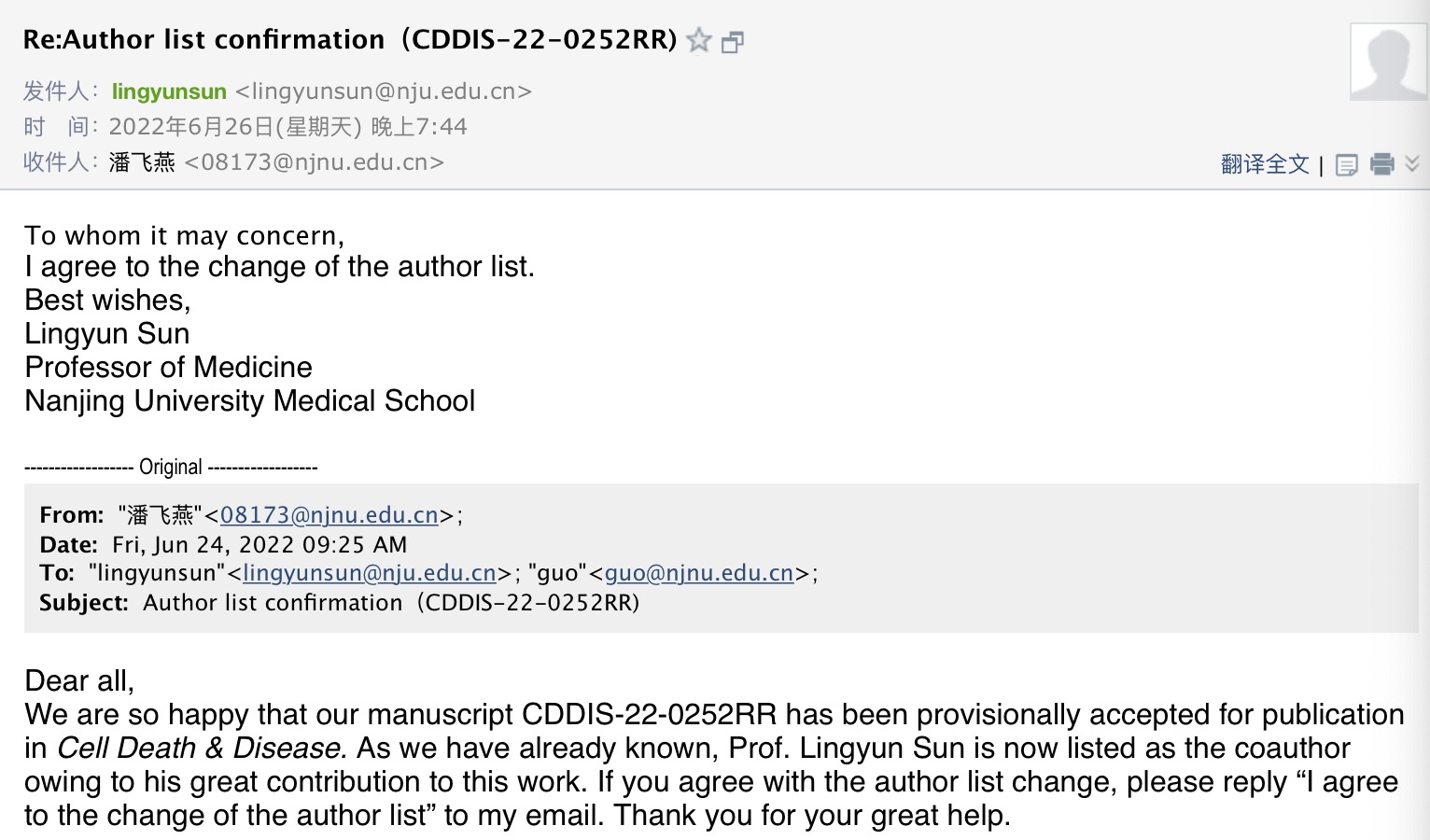


Zhimin Yin


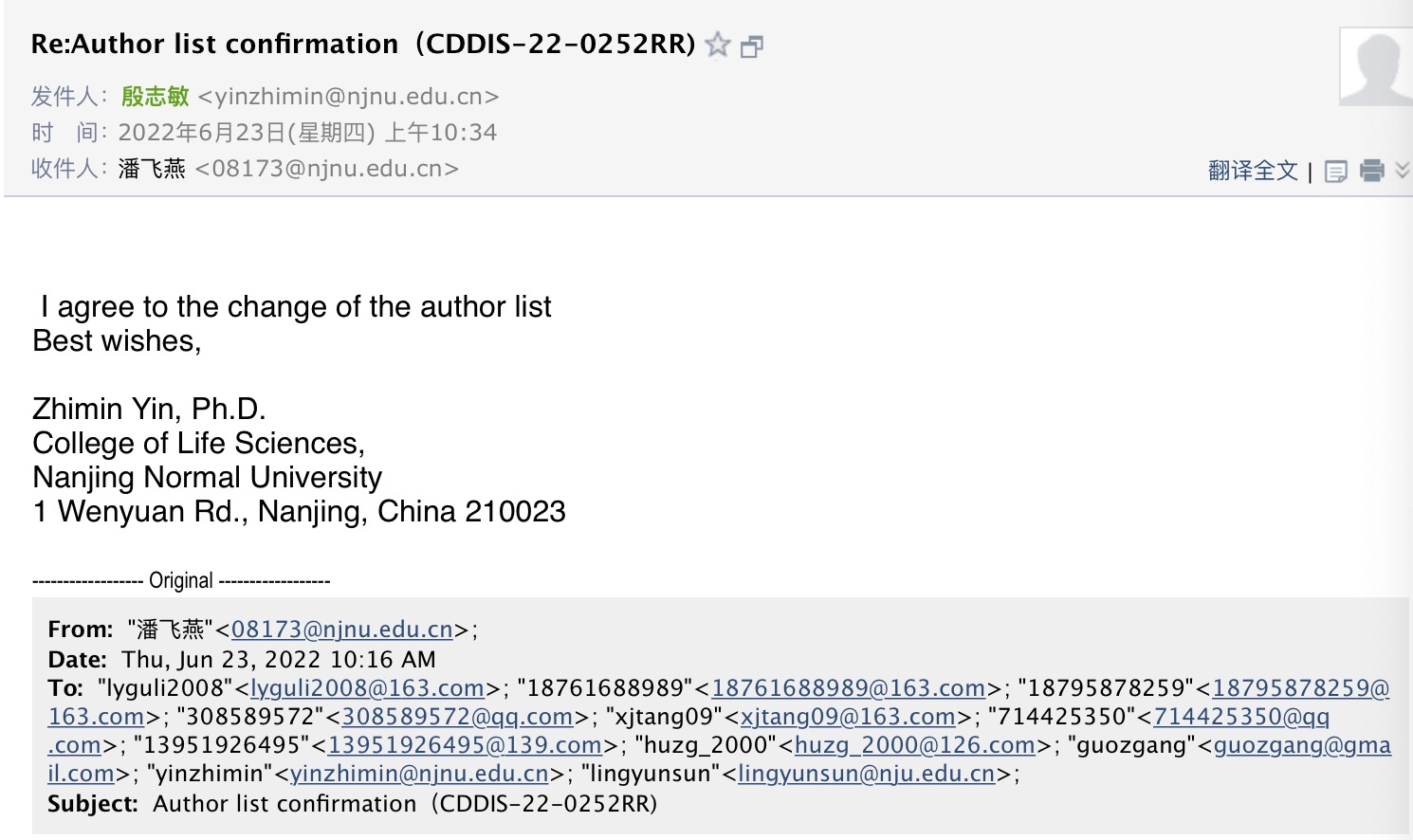


Zhigang Guo


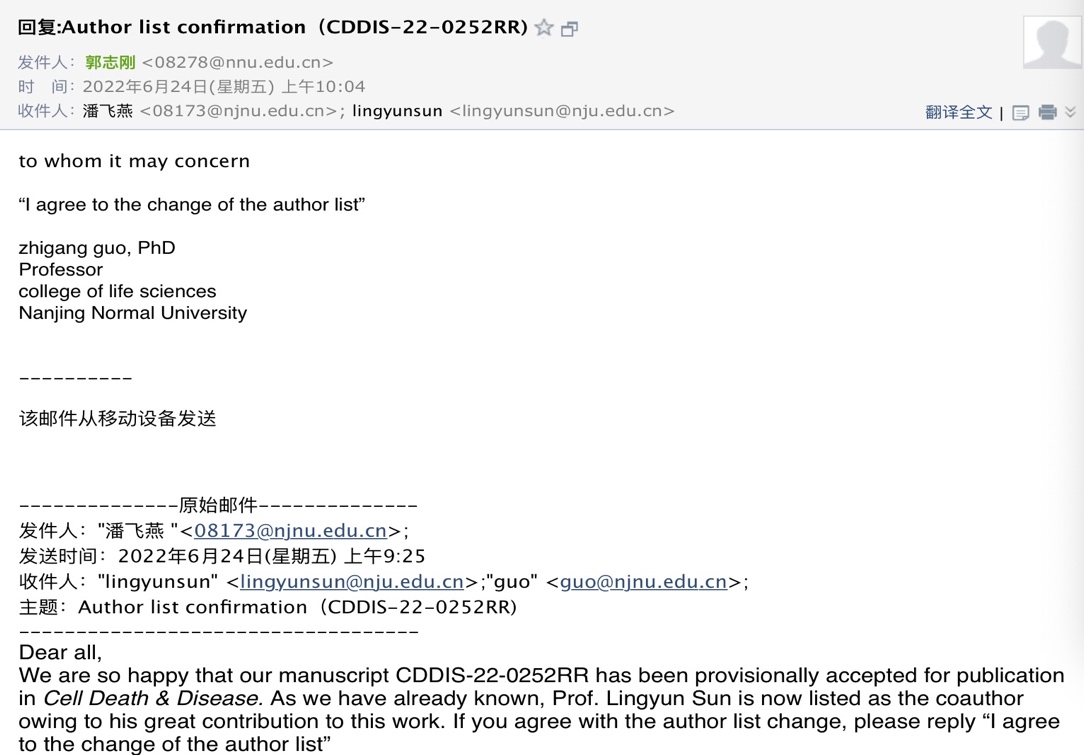

Supplement: Supplementary file 4 — author list confirmation [file 41419_2022_5047_MOESM4_ESM.docx]
